# Catalyst‐Free Collagen Filament Crosslinking for Engineering Anisotropic and Mechanically Robust Tissue Scaffolds

**DOI:** 10.1002/advs.202514319

**Published:** 2025-11-18

**Authors:** JuYeon Kim, Hanjun Hwangbo, ByungJoon Choi, Dogeon Yoon, GeunHyung Kim

**Affiliations:** ^1^ Department of Precision Medicine Sungkyunkwan University School of Medicine (SKKU‐SOM) Suwon 16419 Republic of Korea; ^2^ Hangang Sacred Heart Hospital College of Medicine Hallym University Seoul 07325 Republic of Korea

**Keywords:** anisotropic engineered scaffolds, bioorthogonal crosslinking, collagen hydrogel, wet‐spinning

## Abstract

Engineering mechanically resilient hydrogels from naturally derived proteins, such as collagen and gelatin, remains a key challenge in tissue regeneration, particularly when cell compatibility and structural integrity are simultaneously required. Here, a bioorthogonal crosslinking strategy using rhodamine and polyethylene glycol (PEG) is reported to fabricate dense, mechanically reinforced collagen hydrogels. PEG‐mediated dehydration induces spontaneous peptide bond formation between rhodamine and collagen without the need for additional catalysts, yielding fibrous protein networks with enhanced stiffness. To enable anisotropic tissue engineering, this crosslinking method is integrated with wet‐spinning to produce uniaxially aligned collagen filaments. These constructs exhibit high mechanical strength and support human adipose‐derived stem cell (hASC) encapsulation. Mechanotransductive signaling, including cytoskeletal organization and myogenic gene expression, is effectively activated within the aligned filaments. The applicability of cell‐laden filaments in a murine volumetric muscle loss model is demonstrated, which promoted in vitro differentiation and in vivo functional muscle regeneration. This strategy offers a scalable and cytocompatible platform for generating aligned protein‐based scaffolds with tunable mechanical and biological properties, thereby expanding the toolkit for regenerative medicine.

## Graphical abstract

A bioorthogonal rhodamine/PEG crosslinking strategy is introduced to engineer dense collagen hydrogels with high mechanical resilience and cytocompatibility. Integration with wet‐spinning enables the fabrication of uniaxially aligned, cell‐laden collagen filaments that activate mechanotransductive signaling and support functional muscle regeneration in a murine volumetric muscle loss model.

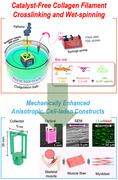

## 1 Introduction

The fabrication of functional tissue constructs that closely replicate the architecture and mechanical properties of native tissues remains a major challenge in regenerative medicine, despite advances in various biofabrication processes.^[^ ^1^, ^2^ ^]^ Protein‐based hydrogels, particularly collagen, gelatin, fibrin, elastin, and decellularized ECMs (dECMs), are widely used owing to their excellent biocompatibility, bioactivity, and ability to promote cell adhesion and proliferation. However, the inherently low mechanical strength of these hydrogels critically limits their applicability for the regeneration of mechanically active or load‐bearing tissues, such as skeletal muscle, myocardium, trachea, and ligament. Although various chemical crosslinking agents—such as tannic acid,^[^ ^3^ ^]^ genipin,^[^ ^4^ ^]^ 1‐ethyl‐3‐(3‐dimethylaminopropyl) carbodiimide (EDC)/N‐hydroxysuccinimide (NHS),^[^ ^5^ ^]^ and multi‐armed polyethylene glycol (PEG)^[^ ^6^ ^]^—have been employed to enhance mechanical stability (Table S1, Supporting Information), achieving sufficient mechanical robustness without compromising cellular viability remains an unmet challenge in the field.

To overcome these challenges, we previously developed a PEG‐assisted dehydration approach that enhances the structural integrity of wet‐spun collagen fibers.^[^ ^7^ ^]^ In this process, the PEG solution functions as a non‐covalent crosslinking agent by inducing dehydration‐driven collagen fibril densification, increasing the fibril packing density, and promoting physical crosslinking. This osmotic‐effect‐based PEG crosslinking of collagen filaments is similar to dehydrothermal treatment, reinforcing collagen integrity without requiring chemical crosslinkers or photopolymerization.

Although PEG‐assisted crosslinking can induce a relatively high draw ratio for wet‐spinning to encourage collagen alignment and strength, the mechanical performance of the fabricated collagen remains insufficient for application in hard tissue regeneration. These results indicated that collagen fibril densification using only PEG did not provide adequate reinforcement compared to chemically crosslinked or composite‐reinforced biomaterials, necessitating additional structural enhancement.

Building on this foundation, we introduce a rhodamine‐assisted crosslinking strategy that forms amide bonds within collagen in a bioorthogonal way, without interfering with cellular processes, and that enhances the mechanical and structural properties of protein‐based filaments. In this method, amide bonds are spontaneously formed between rhodamine and collagen molecules in a PEG‐induced macromolecular crowding environment, which reduces water activity and promotes nucleophilic substitution reactions without external catalysts.^[^ ^8^, ^9^ ^]^ This dual crosslinking mechanism markedly improves filament stiffness and stability while preserving cellular compatibility. Importantly, macromolecular crowding has also been shown to accelerate the gelation of otherwise weakly gelling ECM solutions, thereby improving their printability. For instance, recent studies have shown that macromolecular crowding enables rapid gelation of otherwise weakly gelling ECM solutions, greatly improving their printability.^[^ ^10^ ^]^ The tunable rapid assembly of collagenous elements (TRACE) approach demonstrated that macromolecular crowding with PEG allows direct bioprinting of functional cardiac tissues in ring and tube formats. Additionally, another study showed that macromolecular crowding can stabilize unmodified decellularized ECM solutions from various tissues for extrusion printing with high cell viability.^[^ ^11^ ^]^ These advances highlight the growing importance of macromolecular crowding strategies for fabricating functional ECM‐based constructs.

We initially applied this crosslinking strategy to collagen and demonstrated its capacity to generate highly aligned, mechanically reinforced filaments suitable for supporting cellular activity. To expand the versatility of the platform, we further applied this approach to various protein‐based biomaterials, including gelatin, decellularized extracellular matrix (dECM) derived from porcine cardiac tissue, and gelatin methacryloyl (GelMA), enabling the fabrication of a diverse array of structurally stable, cell‐compatible filaments.

To efficiently enhance filament performance, we incorporated a wet‐spinning process that promotes unidirectional alignment of collagen fibrils and encapsulated cells. Such alignment not only enhances mechanical strength but also facilitates mechanotransductive signaling pathways, driving cytoskeletal reorganization and extracellular matrix remodeling.^[^ ^12^ ^]^ This approach allowed us to precisely control both the structural and cellular architecture of the fabricated filaments.

Combining rhodamine‐assisted crosslinking with wet‐spinning, we established a fabrication platform capable of producing mechanically robust, biologically active, and highly versatile cell‐laden filaments. Using this strategy, we engineered tissue‐mimetic constructs replicating the respective architecture of skeletal muscle, trachea, and multilayered myocardial tissue, with tunable collagen fibril alignment and spatially organized cellular arrangements. In contrast to many existing methods, our approach offers several clear advantages. It promotes covalent bonding between functional groups under mild conditions, allowing for stable collagen crosslinking without the need for harsh chemical treatments. The system is also highly adaptable, capable of incorporating a wide range of small molecules or polymers bearing amine and carboxyl groups, which extends its use beyond collagen‐based materials. The mechanical properties of the constructs can be precisely tuned, ranging from soft to stiff, depending on the application. Moreover, the use of wet spinning enables controlled fiber alignment, closely replicating the anisotropic architecture of native tissues.

Finally, we fabricated collagen‐based human adipose‐derived stem cell (hASC)‐laden muscle constructs and evaluated their regenerative efficacy through comprehensive in vitro and in vivo studies. In a murine volumetric muscle loss (VML) model, the engineered muscle constructs exhibited excellent regenerative performance, promoting functional muscle recovery and extensive muscle fiber regeneration. This work can provide a broadly applicable strategy for engineering mechanically resilient, biologically functional protein‐based scaffolds for regenerative medicine applications across a wide range of tissue types.

## 2 Results and Discussion

### 2.1 Structural and Mechanical Evolution of Collagen Hydrogels through Current Distinct Crosslinking Strategies

**Figure 1:**
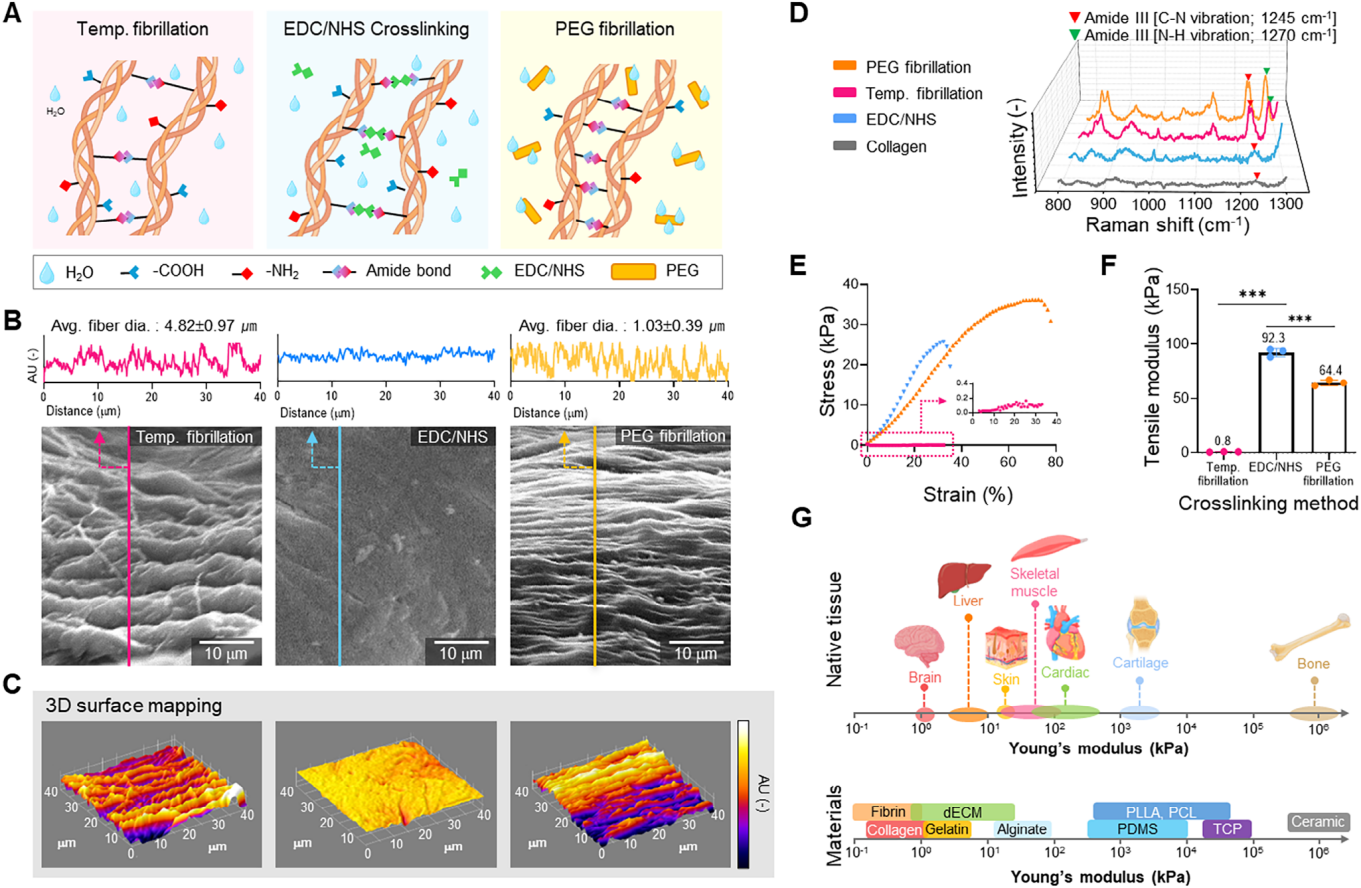
Current typical crosslinking strategies of collagen. A) Schematical illustration, B) scanning electron microscopy (SEM) images, and C) 3D surface mapping of collagen processed using temperature fibrillation, 1‐ethyl‐3‐(3‐dimethylaminopropyl) carbodiimide (EDC)/N‐hydroxysuccinimide (NHS) crosslinking, and polyethylene glycol (PEG) fibrillation. D) Raman spectrum highlighting amide III peaks of various types of crosslinking methods compared with non‐crosslinked collagen. E) Stress–strain curves and F) calculated tensile modulus of various crosslinking strategies (*n* = 3). G) Young's modulus of native tissues, natural polymers, synthetic polymers, and ceramics, illustrating the wide range of stiffness across biological and engineered materials.^[^ ^32^, ^40^ ^]^ Data presented as mean ± standard deviation (SD). Statistical significance was determined using one‐way ANOVA with Tukey's post‐hoc test (^***^ *p*<0.001).

Typical protein‐based hydrogels, such as collagen, gelatin, and dECM, are widely employed in tissue engineering owing to their intrinsic biocompatibility and bioactivity.^[^ ^13^, ^14^, ^15^ ^]^ However, their low mechanical strength necessitates additional crosslinking strategies to enhance structural integrity and mechanical performance, which are critical for supporting cellular behaviors and withstanding physiological forces in vivo.^[^ ^15^ ^]^ To enhance the mechanical properties of collagen‐based hydrogels, widely used strategies include temperature‐induced fibrillation (37 °C incubation) to promote collagen self‐assembly,^[^ ^16^ ^]^ chemical crosslinking via EDC/NHS to establish covalent bonds,^[^ ^17^ ^]^ and PEG‐assisted dehydration^[^ ^7^ ^]^ to densify fibrillar networks through hydrogen bonding (**Figure** **1**A).

These crosslinking strategies using 3 wt.% of collagen not only enhanced the mechanical properties but also imparted distinct topographical features to the fibrous collagen structures (Figure 1B,C; Figure S1, Supporting Information). The morphology of collagen fibrils varies significantly depending on the crosslinking method used. During temperature‐induced fibrillation, collagen self‐assembles into a loose but aligned network. For temperature‐induced fibrillation, scanning electron microscopy (SEM) and topographical imaging revealed groove‐like morphologies with visible interfibrillar spaces and minimal fiber bundling, reflecting natural fibrillogenesis. However, EDC/NHS crosslinking formed a highly dense and random nanofibril network. The images appeared smoother and more consolidated than in temperature‐induced fibrillation, but the fibril diameters tended to be slightly smaller (50–150 nm). This reduction may be attributed to the inhibition of lateral fibril growth during crosslinking as EDC/NHS covalently bonds adjacent collagen molecules, effectively “locking in” the microstructure at an early stage of fibrillogenesis.^[^ ^18^ ^]^ Moreover, PEG‐induced dehydration showed markedly different characteristics. The images revealed highly aligned fibrillar bundles, indicating substantial lateral aggregation. This morphology likely arises from macromolecular crowding induced by PEG‐driven osmotic dehydration, which concentrates collagen molecules and accelerates fibril formation, yielding denser bundles than temperature‐induced fibrillation.^[^ ^6^ ^]^

In addition, Raman spectroscopy was used to compare the molecular changes in collagen induced by thermal fibrillation at 37 °C, EDC/NHS crosslinking, and PEG‐induced fibrillation with that in untreated collagen, with a particular emphasis on amide bond‐associated peaks (Figure 1D). Notably, the amide III doublet (red: C─N vibration, 1245 cm^−1^; green: N─H vibration, 1270 cm^−1^) reflects collagen secondary structure, with the ≈1242 cm^−1^ peak indicating disordered regions and the ≈1270 cm^−1^ peak corresponding to ordered collagen fiber.^[^ ^19^, ^20^, ^21^ ^]^

Both PEG‐treated and thermally fibrillated collagen samples exhibited a marked increase in intensity at ≈1270 cm^−1^, indicating enhanced triple‐helix alignment relative to untreated and EDC/NHS‐crosslinked collagen. This spectral shift reflects the effective promotion of collagen self‐assembly into fibrillar structures facilitated by the macromolecular crowding effect of PEG. Compared to pure collagen, all three treatments induced spectral changes indicative of improved structural organization. Along with morphological changes, mechanical testing (Figure 1E,F) showed that EDC/NHS crosslinking yielded the highest reinforcement via covalent interchain bonds, while PEG dehydration provided intermediate stabilization through non‐covalent fibrillar compaction. Thermal fibrillation resulted in the lowest mechanical enhancement.

Despite employing various crosslinking strategies to reinforce collagen‐based bioconstructs, the mechanical properties achieved remain substantially inferior to those required for load‐bearing tissue regeneration. PEG‐mediated physical crosslinking and EDC/NHS‐mediated chemical crosslinking enhanced the tensile moduli of collagen constructs to ≈0.6 and 0.9 kPa, respectively, surpassing that of thermally fibrillated collagen (Figure 1F). However, comparing the Young's moduli of various biomaterials and native tissues indicates that the required modulus of crosslinked collagen is still insufficient to replicate the mechanical environment of many target tissues, such as skeletal/cardiac muscle or cartilage (Figure 1G). For example, the Young's modulus of native skeletal muscle tissue is 20–100 kPa, which is several orders of magnitude higher than that of physically or chemically crosslinked collagen constructs. This mechanical disparity emphasizes the importance of advanced crosslinking methods for collagen or collagen‐based hydrogels (such as gelatin and dECM) to meet the diverse mechanical requirements of engineered tissues in regenerative medicine.

### 2.2 Fibrous Collagen Structure through PEG/Rhodamine‐Facilitated Crosslinking and Wet‐Spinning

**Figure 2:**
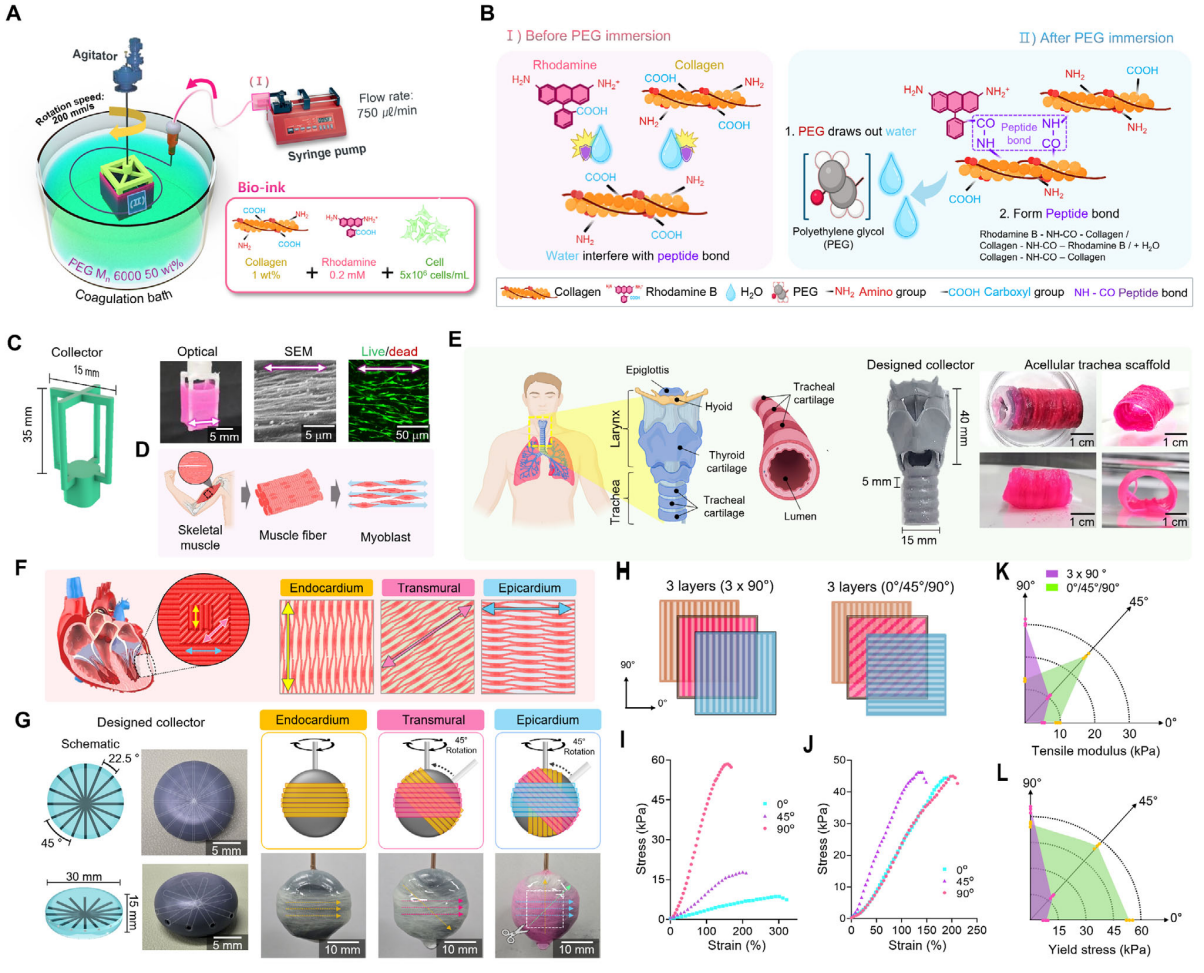
Polyethylene glycol (PEG)/rhodamine‐assisted crosslinking combined with wet‐spinning enables the fabrication of mechanically robust and uniaxially aligned collagen‐based cell constructs. Schematic illustrations of (A) the wet‐spinning process of rhodamine‐incorporated collagen bioink and B) the peptide bond formation of the rhodamine‐assisted PEG fibrillation mechanism. C) 3D model and optical image of the collector, and live (green)/dead (red) images of C2C12 cells processed using rhodamine‐assisted crosslinking of PEG‐dehydrated collagen filaments. D) Schematic illustration of anisotropic organization of skeletal muscle tissue. E) Schematic illustration of native human tracheal cartilage as a design reference, along with optical images of the modified collector and the resulting acellular tracheal scaffold. F) Schematic diagram of the myocardium alignment and G) wet‐spinning process using modified collectors able to rotate to form fiber layers with various orientations. H) Schematics of multilayered collagen constructs illustrating two architectures: an anisotropic structure (3 × 90°) and an alternating structure (0°/45°/90°). Stress–strain curves of (I) the anisotropic structure (3 × 90°) and J) the alternating structure (0°/45°/90°) under uniaxial tension applied at 0°, 45°, and 90°. Quantitative analyses of (K) tensile modulus (*n* = 3) and L) yield stress for each orientation (*n* = 3).

To overcome the poor mechanical properties of collagen‐based hydrogels, we introduce an approach that enhances the mechanical performance of low‐concentration collagen hydrogels by promoting covalent bonding between rhodamine and collagen functional groups, facilitated by PEG‐mediated dehydration. Subsequently, we utilized a wet‐spinning process to align the collagen fibers, further augmenting not only the mechanical strength but also the topographical cues of the engineered constructs (**Figure** **2**A; Movie S1, Supporting Information). The crosslinking mechanism can be described as peptide bond formation between the amine and carboxyl groups of rhodamine and collagen (Figure 2B). This reaction occurs under room temperature, ensuring the preservation of bioactivity and structural integrity of the collagen while improving its mechanical properties.

A bioink solution composed of 1 wt.% collagen and 0.2 mm rhodamine was extruded into a PEG‐based coagulation bath (50 wt.%) under controlled flow rate (750 µL min^−1^) and rotational speed (200 mm s^−1^) of the collector. Figure 2C presents the optical, live (green)/dead (red) images of hASCs and SEM images of the fabricated collagen structure with aligned fibrils. This collagen architecture and alignment can significantly enhance the mechanical properties and closely mimic the native ECM.

The versatility of the wet‐spinning process was further demonstrated by tailoring the geometry of the collector; we fabricated collagen constructs with diverse alignments for tissue‐specific applications. For skeletal muscle engineering, unidirectionally aligned cell‐laden constructs were produced to mimic native muscle fiber architecture (Figure 2D). Similarly, a trachea‐shaped collector enabled circular collagen alignment replicating the human tracheal structure (Figure 2E). To recapitulate the anisotropic architecture of cardiac tissue, a rotating collector was employed to generate fibrous constructs with spatially controlled fiber orientations, inspired by the transmural fiber alignment of the myocardium, which underlies its twisting contraction (Figure 2F,G). By adjusting the collector rotation, multilayered collagen constructs with distinct fiber orientations (0°, 45°, 90°) were fabricated (Figure 2G; Figures S2 and S3, Supporting Information). To mimic the multilayered organization of myocardial tissue, two architectures were generated: an anisotropic structure (3 × 90°) and an alternating structure (0°/45°/90°). Mechanical testing (Figure 2I,J) revealed that the anisotropic structure exhibited high tensile strength along the fiber alignment but showed a reduced modulus as the loading direction approached perpendicular. In contrast, the alternating structure displayed a symmetric response, maintaining relatively high yield stress across multiple orientations (Figure 2K,L). These results demonstrate that controlled fiber orientation significantly influences the mechanical anisotropy of the constructs, and that alternating architectures better capture the multidirectional contractile behavior of native myocardium.

### 2.3 Non‐Catalytic Crosslinking of Collagen via PEG Dehydration and Organic Molecules

**Figure 3:**
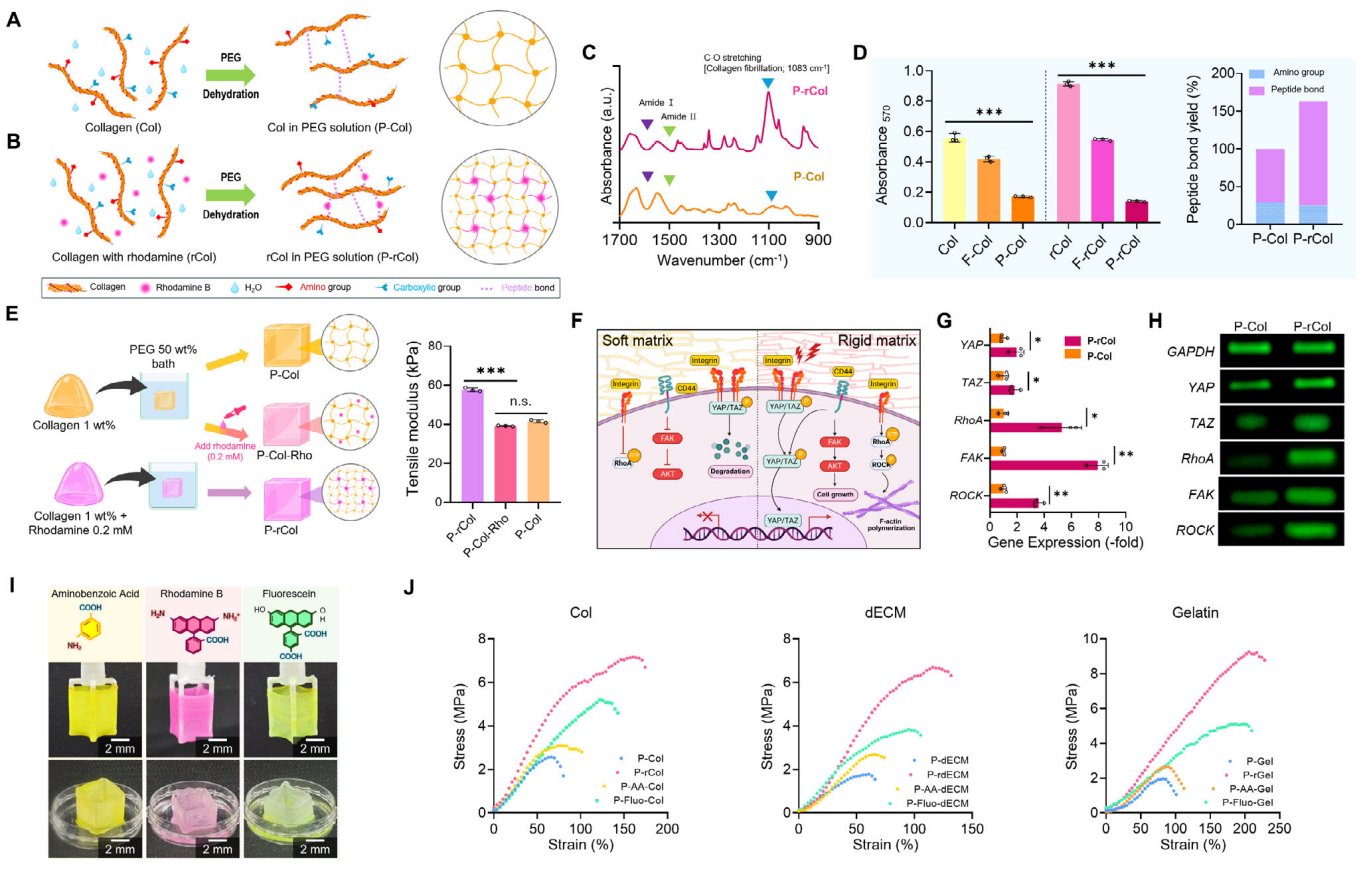
Structural and functional analyses of collagen (Col) fibrils crosslinked by polyethylene glycol (PEG) and rhodamine. Schematic diagrams illustrating peptide bond formation during PEG‐induced dehydration of Col fibers A) without and B) with rhodamine. C) Fourier‐transform infrared spectroscopy comparing P‐Col and P‐rCol, highlighting changes in chemical bonding. D) Ninhydrin assay results showing absorbance values for Col, thermally fibrillated collagen (F‐Col), P‐Col, and their rhodamine‐incorporated counterparts, along with quantification of peptide bond yield in P‐Col and P‐rCol (*n* = 3). E) Schematic illustration of the fabrication process, accompanied by quantified tensile modulus for P‐Col, P‐Col‐Rho, and P‐rCol structures (*n* = 3). F) Diagrams of mechanotransduction‐related intercellular signaling pathways, with corresponding G) gene expression analyses (*n* = 3) and H) agarose gel electrophoresis of PCR products from C2C12 cells cultured on P‐Col and P‐rCol after three days. I) Optical images of collagen structures integrated with organic molecules bearing both carboxylic and amide functional groups (Aminobenzoic Acid, Rhodamine, and Fluorescein). J) Stress–strain curves comparing Col‐, dECM‐, and gelatin‐based hydrogels incorporating these functional molecules. Data presented as mean ± standard deviation (SD). Statistical significance for three or more treatment groups, one‐way analysis of variance (ANOVA) was conducted, followed by Tukey's post‐hoc test. Student's t‐test was used to compare two groups (n.s. = statistical nonsignificance, ^*^ *p*<0.05, ^**^ *p*<0.01, ^***^ *p*<0.001).

The schematics shown in **Figure** **3**A,B indicate the molecular organization of collagen dehydrated with PEG solution (P‐Col) and collagen crosslinked with rhodamine under PEG solution (P‐rCol), respectively. Native collagen molecules exhibited a disordered and loosely bound network owing to low intermolecular interactions. However, when the collagen molecules were immersed in the PEG solution, P‐Col was formed because of an increased fibril packing density (Figure 3A). In contrast, denser collagen fibrils were formed if the rhodamine could be premixed with collagen hydrogel and then immersed in the PEG solution. This reaction mechanism to form the P‐rCol can be explained by considering the behavior of the collagen/rhodamine mixture in the PEG bath. Under normal aqueous conditions, the reactive groups of rhodamine and collagen do not readily form peptide bonds because of their highly hydrated shells and lack of coupling agents. However, introducing the collagen hydrogel into a concentrated PEG solution can trigger a dehydration‐driven peptide bond reaction.^[^ ^7^, ^22^ ^]^ PEG acts as a macromolecular crowding and osmotic agent, pulling water molecules out of the collagen fibrils, thereby decreasing local water activity. This dehydration significantly reduces the solvation shell surrounding the functional groups and increases their effective molarity and reactivity. Consequently, the reactive groups of rhodamine and collagen form peptide bonds through nucleophilic substitutions. This reaction can be facilitated by the entropic forces associated with PEG‐induced crowding, which mimic the intracellular environment and promote spontaneous condensation, demonstrating that PEG‐mediated crowding environments can accelerate peptide bond formation, polymerization, and bioconjugation through enhanced molecular proximity and reduced hydration.^[^ ^23^, ^24^ ^]^

The mechanism is qualitatively demonstrated in Figure S4 (Supporting Information), where the collagen/rhodamine hydrogels were immersed in distilled water and 50 wt.% PEG solution. A dissolved hydrogel was observed within 30 s in distilled water, indicating poor mechanical stability owing to the absence of peptide bonding. In contrast, the collagen/rhodamine filament maintained distinct shape stability when immersed in the PEG solution, confirming that the PEG‐induced dehydration process facilitated the bonding between collagen and rhodamine, thereby enhancing the mechanical stability of the hydrogel. In addition to simply comparing the densified P‐Col and P‐rCol fibrils, the SEM images revealed that P‐rCol forms compact, homogenous fibrils with a diameter of 0.6 µm, whereas P‐Col maintains a non‐homogenous and irregular fibrous morphology (diameter = 2.2 µm; Figure S5, Supporting Information).

Fourier‐transform infrared (FTIR) spectroscopy was used to assess biochemical modifications induced by crosslinking (Figure 3C). Both samples exhibited characteristic collagen bands—amide I (≈1650 cm^−1^, C═O stretching) and amide II (≈1550 cm^−1^, N─H bending and C─N stretching). Notably, the P‐rCol sample showed an enhanced peak at 1083 cm^−1^, indicative of increased intermolecular interactions within the collagen network.^[^ ^25^ ^]^ The enhanced 1083 cm^−1^ peak can suggest significant alterations in the molecular environment of collagen, resulting from its interaction with PEG and the rhodamine. These modifications indicate successful chemical conjugation, which is further supported by new amide bond formation. Together, these spectral changes confirm that PEG and rhodamine treatment not only alter the structural organization of collagen but also facilitate covalent attachment, enhancing the functional properties of the resulting material.

Circular dichroism (CD) spectroscopy was employed to analyze the secondary structure of collagen, with particular focus on the changes to α‐helical (triple helical) structure during fibrillation (Figure S6, Supporting Information). The collagen hydrogel exhibited a distinct positive peak at 222 nm, indicative of a well‐preserved triple helical structure.^[^ ^26^ ^]^ In contrast, PEG‐fibrillated collagen (P‐Col) showed a noticeable reduction in peak intensity at 222 nm, suggesting partial disruption of the triple helix due to fibril formation. This effect was further amplified by the incorporation of rhodamine (P‐rCol), which led to an even greater decrease in ellipticity. These results are consistent with findings by Drzewiecki et al., who reported that unfibrillated collagen exhibits a strong positive ellipticity near 222 nm, which diminishes and becomes less defined as fibrillogenesis progresses.^[^ ^27^ ^]^

The availability of amine groups after rhodamine incorporation was evaluated by ninhydrin assay (Figure 3D). rCol (pure mixture of collagen and rhodamine) exhibited higher absorbance than pure collagen (Col), reflecting additional amine groups introduced by rhodamine. In thermally fibrillated samples (F‐Col and F‐rCol), amine group content decreased, suggesting consumption through collagen–collagen and collagen–rhodamine peptide bond formation. The most pronounced reduction was observed in P‐Col and P‐rCol, indicating extensive amide bond formation driven by PEG‐induced dehydration. These results demonstrate that the ninhydrin assay can effectively assess peptide bond formation within the hydrogel matrix, and that both rhodamine incorporation and PEG treatment promote covalent crosslinking.^[^ ^28^ ^]^

Moreover, mechanical testing demonstrated the direct impact of these structural changes (Figure 3E; Figure S7, Supporting Information). P‐rCol exhibited significantly higher tensile modulus and strength than P‐Col, as indicated by the stress–strain curves. This enhancement results from increased peptide bonding, improved molecular alignment, and fibril homogeneity, which contribute to effective load transfer and mechanical resilience.^[^ ^29^, ^30^ ^]^ Particularly, the importance of fibrillar homogeneity has been further supported by demonstrating that uniform collagen fiber networks reduce stress concentrations and increase fatigue resistance. Homogeneous fibrils also maintain stable hydration profiles by preventing mechanical weakening owing to swelling or collapse. In contrast, non‐homogeneous fibrils, typified by P‐Col, show irregular crosslinking and variable diameters, leading to early mechanical failure and reduced durability.

Furthermore, peptide bonding in P‐rCol not only enhanced hydrogel mechanical strength but also promoted uniform collagen fibril formation, potentially influencing cell behavior via mechanotransduction (Figure 3F). To assess this effect, C2C12 (5 × 10⁶ cells mL^−1^) were encapsulated, and early mechanosensitive gene expression was analyzed on day 3. Both quantitative and qualitative expression of *YAP, TAZ, RhoA, FAK*, and *ROCK* were significantly elevated in P‐rCol‐treated cells compared to P‐Col (Figure 3G,H). Notably, *FAK* and *RhoA* exhibited pronounced upregulation, likely driven by the increased stiffness and homogeneous architecture of the collagen matrix. As illustrated in the mechanotransduction pathway schematic (Figure 3F), enhanced matrix stiffness promotes cell–ECM adhesion and FAK activation at focal adhesions, which subsequently stimulates RhoA‐mediated cytoskeletal tension and contractility.

To further validate the role of amide bond formation in enhancing the mechanical properties of collagen through PEG‐induced dehydration, we used two additional organic molecules, aminobenzoic acid (AA) and fluorescein (Fluo), which possess both amine and carboxyl functional groups capable of participating in peptide bonding reactions (Figure 3I). In addition, these molecules have been incorporated into various collagen‐based hydrogels, including collagen, porcine muscle‐derived dECM, and gelatin (Gel), thus replacing rhodamine in the system.

To indirectly demonstrate the peptide reaction of the hydrogels, a tensile stress–strain test was performed. The mechanical testing results showed that both AA‐ and Fluo‐treated hydrogels exhibited enhanced tensile properties than untreated hydrogels (Figure 3J). These results support the hypothesis that PEG‐induced dehydration promotes amide bond formation between collagen‐based hydrogels and amine‐ and carboxyl‐containing compounds, resulting in fibril densification and matrix stiffening. Based on these results, we carefully estimate that this method can present a broadly applicable chemical framework for enhancing the mechanical properties of various collagen‐derived biomaterials using small‐molecule crosslinkers in combination with a PEG dehydration‐driven process without toxic effects.

### 2.4 Tunable Stiffness and Alignment of Collagen Microfilaments through PEG/Rhodamine‐Assisted Spinning

**Figure 4:**
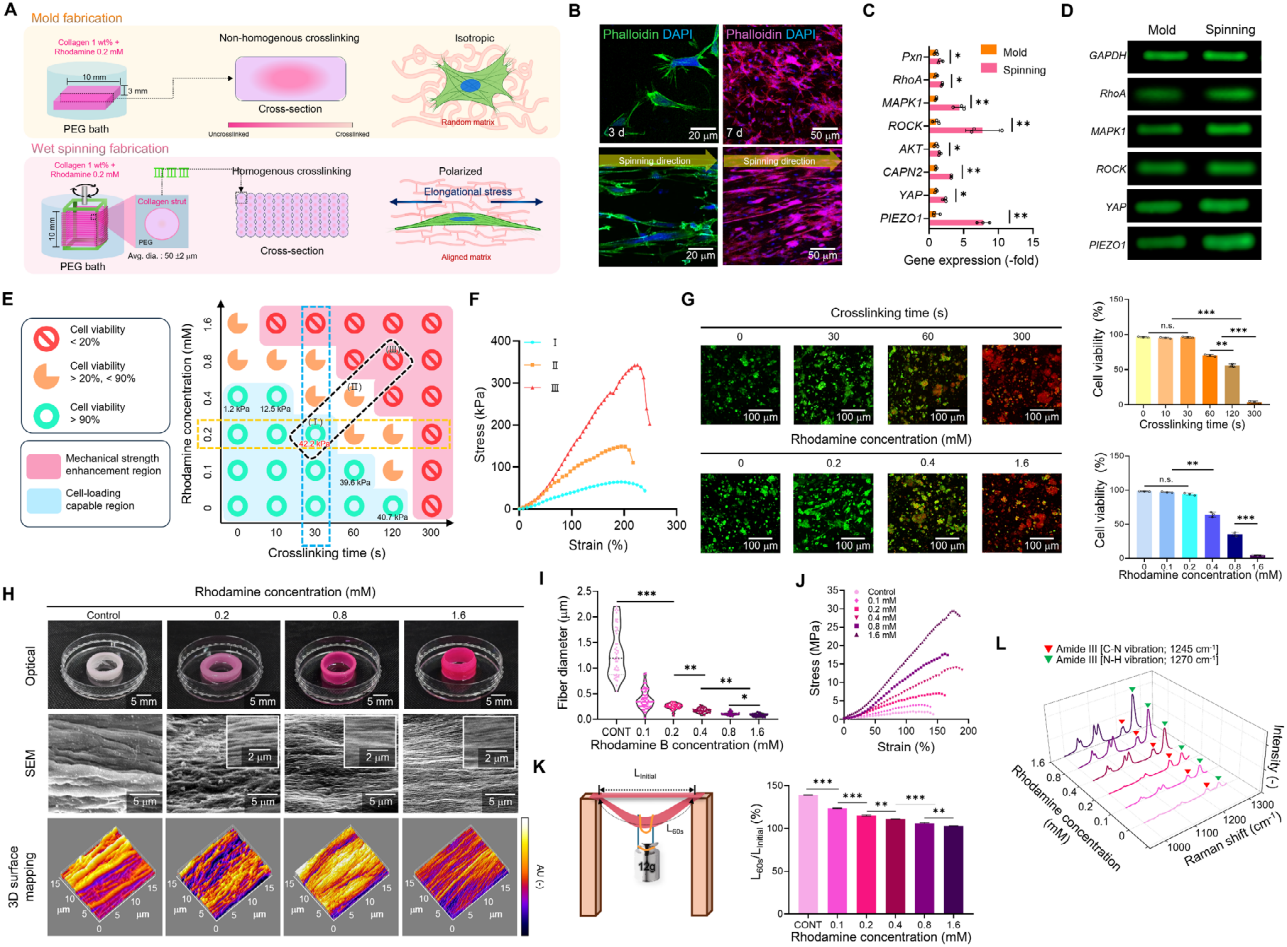
Rhodamine‐mediated wet‐spinning modulating collagen fiber morphology, mechanics, and mechanotransductive cell responses. A) Schematic illustration and B) DAPI/phalloidin‐stained images of C2C12 cells encapsulated in rhodamine‐incorporated collagen processed by molding or wet‐spinning in a polyethylene glycol (PEG) bath, demonstrating F‐actin alignment and cellular stretching. C) Gene expression levels of mechanotransduction‐related markers (*n* = 3). D) Agarose gel electrophoresis of PCR products from C2C12 cells cultured in collagen constructs processed via molding or spinning. E) Process diagram showing the effects of rhodamine concentration and crosslinking duration; numbers within the graph represent the tensile modulus of each structure. F) Mechanical properties are categorized into three regimes: (I) stable cell survival, (II) partial cell death, and (III) non‐viable conditions. G) Live/dead staining and quantified viability of C2C12 cells subjected to different rhodamine concentrations and crosslinking times (*n* = 3). H) Optical images, scanning electron microscopy (SEM) micrographs, and 3D surface mapping of collagen fibers processed with 0–1.6 mm rhodamine. I) Measured fiber diameter (*n* = 50) and J) stress–strain curves of collagen constructs produced with varying rhodamine concentrations. K) Schematics of hanging weight test and quantified L_60_/L_initial_ ratios for collagen constructs processed with varying rhodamine concentrations (*n* = 3). L) Raman spectra of rhodamine‐processed collagen samples, highlighting changes in chemical structure. Data presented as mean ± standard deviation (SD). Statistical significance for three or more treatment groups, one‐way analysis of variance (ANOVA) was conducted, followed by Tukey's post‐hoc test. Student's t‐test was used to compare two groups (n.s. = statistical nonsignificance, ^*^ *p*<0.05, ^**^ *p*<0.01, ^***^ *p*<0.001).

In the previous section, we demonstrated that dehydrating the collagen/rhodamine bioink with PEG significantly enhanced the mechanical strength of the resulting hydrogel constructs. However, this bioink rapidly dehydrated when directly immersed in a PEG solution, primarily only on the outer surface of the construct (**Figure** **4**A). This leads to localized and dense peptide bond formation at the periphery, whereas the core region remains insufficiently crosslinked owing to limited PEG penetration. Therefore, the final bulk hydrogel exhibited a non‐homogeneous crosslinking architecture with a strong outer layer and weak inner core, which may compromise the structural integrity and uniform cellular responses. To overcome this limitation, we employed a wet‐spinning technique to fabricate collagen microfilaments (lower schematic, Figure 4A). The hydrogel underwent uniform dehydration along the filament axis by continuous extrusion of the collagen/rhodamine bioink into a PEG bath. This wet‐spinning process enabled consistent PEG diffusion and evenly distributed amide bond formation throughout the filament structure. This can effectively resolve the uneven crosslinking observed during bulk hydrogel immersion and produce a mechanically robust filamentous cell construct.

Furthermore, the stretching process during wet‐spinning offers key advantages by providing topographical and mechanical cues that promote cellular alignment and tissue organization (Figure 4A). Fluorescence images showed that encapsulated C2C12 (1 wt.% and 5 × 10^6^ cells mL^−1^) cultured on non‐stretched, mold‐cast P‐rCol exhibited random cell orientations on day 7, with limited cytoskeletal organization (Figure 4B). In contrast, the cells on wet‐spun stretched P‐rCol (S‐P‐rCol) demonstrated relatively enhanced alignment along the stretching direction, as indicated by the elongated F‐actin and nuclei on day 7 (Figure 4B; Figutre S8, Supporting Information). This alignment was quantified by a radial plot, where a higher directional coherence was observed in S‐P‐rCol than in mold‐cast P‐rCol. Moreover, mechanical stimuli during wet‐spinning can activate intracellular signaling pathways regulating cytoskeletal remodeling and cell orientation. Mechanotransduction‐related gene expressions were examined to evaluate the effects of mechanical stimulation on cells. Genes such as *Pxn* (focal adhesion signaling), *RhoA/ROCK* (cytoskeletal tension control), *MAPK1* (tissue remodeling signaling), *AKT* (cell survival and cytoskeletal stability), *CAPN2* (cytoskeletal remodeling protease), *YAP/TAZ* (cell proliferation and differentiation regulation), and *PIEZO1* (mechanosensitive calcium channel) were upregulated in the spinning group, with *ROCK* and *PIEZO1* showing particularly pronounced increases (Figure 4C,D). The results indicate that the wet‐spinning process not only achieves uniform crosslinking and mechanical integrity but also facilitates cell alignment through mechanical stimulation, demonstrating the feasibility of fabricating engineered anisotropic tissues such as muscles, tendons, or nerves.

Figure S9A (Supporting Information) presents a schematic of the wet‐spinning system, where a collagen bioink (1 wt.% and 5 × 10^6^ C2C12 cells mL^−1^) containing 0–1.6 mm rhodamine was extruded into a rotating collector in a bath filled with 50 wt.% PEG solution. Through this process, the influence of rhodamine concentration and crosslinking duration on cell viability and the impact of fiber processing on mechanical strength were analyzed. First, a process diagram under the fixed conditions (collagen flow rate: 750 µL min^−1^, rhodamine: 0.2 mm, PEG concentration: 50 wt.%) showed proper fiber formation region across 0.1–2% wt.% collagen and 50–300 mm s^−1^. Each symbol shows the fiber formation quality (×: fracture, O: fiber, Δ: bead), and the results demonstrate that successful fiber formation depends on a delicate balance (high rotational speed can induce high elongational stress, resulting in collagen filament fracture) between collagen concentration and rotation rate (Figure S9B, Supporting Information).

After selecting an appropriate processing region (1 wt.% collagen and 200 mm s^−1^) for filament formation, the effect of rhodamine and crosslinking duration on the viability of C2C12 cells laden in the collagen filaments was observed.

Figure 4E presents a process diagram showing the relationship between crosslinking time, rhodamine concentration, and cell viability. The tested conditions were grouped into three categories: I) stable cell survival, II) partial cell death, and III) non‐viable conditions. As shown in Figure 4F and Figure S10 (Supporting Information), increasing rhodamine concentration and extending crosslinking time led to notable improvements in mechanical properties. However, excessive rhodamine loading (>0.2 mm) and prolonged retention times (>30 s) significantly reduced cell viability (Figure 4G; Figure S11, Supporting Information). This phenomenon occurs because high concentrations of rhodamine, particularly in its free form, can disrupt cell membranes, generate reactive oxygen species, and interfere with mitochondrial activity, thereby reducing metabolic activity and cell survival.^[^ ^31^ ^]^ Similarly, increasing the wet‐spinning retention time in the PEG solution, which directly correlates with the extent of crosslinking, also reduces cell viability. As seen in the results, cell viability gradually decreased over 30 s because the residual stress caused by the dehydrated collagen fibrils and the peptide bond reaction could induce significant damage to the cells intercalated between the collagen fibrils, eventually reducing cell viability. A similar phenomenon was observed in the collagen dehydration process in PEG solution in our previous study.^[^ ^7^ ^]^

Additionally, Figure S12A (Supporting Information) shows SEM images comparing stretched P‐Col (S‐P‐Col) and P‐rCol (S‐P‐rCol) filaments after the wet‐spinning process with the materials (collagen: 1 wt.% and rhodamine: 0.2 mm) and processing conditions (collagen flow rate: 750 µL min^−1^, PEG concentration: 50 wt.%, rotation speed: 200 mm s^−1^). The collagen fibrils of S‐P‐rCol were more tightly packed and had a smaller fibril diameter (0.37±0.12 µm) than those of S‐P‐Col (1.12±0.32 µm), indicating enhanced mechanical properties (Figure S12B, Supporting Information). Figure S12C (Supporting Information) shows the tensile stress–strain behaviors of the S‐P‐Col and S‐P‐rCol structures. S‐P‐rCol exhibited significantly better mechanical properties than other samples. This enhancement can be attributed to the 1) wet‐spinning process and efficient collagen fibril alignment in the direction of the collector, and 2) PEG‐induced peptide bonds in the S‐P‐rCol hydrogel, promoting denser molecular packing and crosslinking, and stabilizing the network mechanically.

Additionally, surface optical images and low‐magnification SEM revealed the layered architecture of the constructs, formed by the stacking of deposited collagen filaments (Figure S13, Supporting Information). The close packing of filaments during collection in the PEG bath resulted in dense, sheet‐like assemblies without visible gaps or delamination, supporting the structural integrity of the constructs. Furthermore, the degradation profile of S‐P‐rCol showed stable mass retention for up to 16 days in PBS (Figure S14A, Supporting Information). In contrast, exposure to a 10 U mL^−1^ collagenase solution led to rapid degradation, with complete breakdown observed by day 12. During enzymatic degradation, the constructs exhibited a marked decline in mechanical strength (Figure S14B,C, Supporting Information).

We have previously reported the appropriate rhodamine concentration and crosslinking duration that allowed high cell viability when using the wet‐spinning method with cell‐laden collagen/rhodamine bioinks. In this section, we focus on evaluating the mechanical performance of acellular constructs comprising 1 wt.% collagen to assess the maximum mechanical properties achievable under the defined fabrication parameters. As shown in the image, all constructs were fabricated using the same wet‐spinning conditions (collagen flow rate: 750 µL min^−1^, PEG concentration: 50 wt.%, rotation speed: 200 mm s^−1^) and PEG bath crosslinking duration (300 s), with only the rhodamine concentration varied across 0–1.6 mm (Figure S15A, Supporting Information).

Figure 4H and Figure S15B (Supporting Information) present optical images, SEM micrographs, and 3D surface maps of collagen constructs fabricated via wet spinning with varying rhodamine concentrations incorporated into the bioink. The SEM images revealed that the collagen fibers in the rhodamine‐crosslinked constructs exhibited a more aligned and densely packed morphology than those in the control. This enhanced fiber alignment was further confirmed by 3D surface mapping, which showed a dominant anisotropic fiber direction in the crosslinked groups (Figure 4H). SEM analysis revealed that both fibril diameter and size heterogeneity decreased progressively with increasing rhodamine content, indicating the formation of dense and compact collagen fibrillar networks (Figure 4I; Figure S15B, Supporting Information). This morphological structure could result from the homogeneous and increased formation of amide bonds between rhodamine and collagen under PEG‐induced dehydration, leading to robust physical and chemical crosslinking.

Mechanical properties were assessed by tensile testing (Figure 4J; Figure S15C–E, Supporting Information). The stress–strain curves demonstrated a concentration‐dependent enhancement in mechanical stiffness with increasing rhodamine content in the collagen bioink. At 1.6 mm rhodamine concentration, the tensile modulus of the collagen structure reached ≈12 ± 0.05 MPa, which is comparable to the Young's modulus of cartilage.^[^ ^32^ ^]^ To further assess structural stability, we performed optical imaging of ring‐shaped constructs (Figure S16A, Supporting Information). Rhodamine‐assisted samples exhibited more defined circularity and greater structural height with increasing rhodamine concentration, supporting enhanced mechanical integrity and improved shape fidelity observed in tensile testing (Figure S16B,C, Supporting Information).

A hanging weight test (12 g) qualitatively supported the mechanical properties, demonstrating improved load‐bearing capacity in constructs with higher rhodamine concentrations. As expected, the quantified displacement also decreased as the rhodamine content increased, emphasizing improved resistance to mechanical deformation (Figure 4K; Figure S17, Movie S2, Supporting Information).

FTIR spectral analysis revealed that the C─O stretching peak (collagen fibrillation‐related; highlighted in blue) became more pronounced with increasing rhodamine concentrations (Figure S18, Supporting Information).^[^ ^25^ ^]^ This trend was consistent with a gradual increase in the amide III peak (red: C─N vibration, 1245 cm^−1^; green: N─H vibration, 1270 cm^−1^) observed in the Raman spectra, further supporting the same pattern (Figure 4L). These findings indicate that, in addition to the characteristic collagen peaks, the increased absorbance intensity in regions corresponding to intermolecular interactions confirms successful chemical crosslinking facilitated by rhodamine. Collectively, these results suggested that rhodamine‐mediated crosslinking significantly improved the structural organization, mechanical strength, and molecular stability of collagen constructs. These results demonstrate that collagen‐based structures with significantly enhanced stiffness may be fabricated by modulating the collagen concentration, crosslinking period, and wet‐spinning parameters within the collagen/rhodamine/PEG processing system. Such tunability in mechanical properties may provide a versatile platform for tailoring collagen constructs to match the mechanical requirements of soft tissues, such as the skin, to load‐bearing tissues, such as bone.

### 2.5 Application of Wet‐Spun Cell‐Laden Collagen Filaments for Muscle Regeneration

In this study, we compared the effects of conventional bioprinted cell‐structure and S‐P‐rCol cell filament on myogenic differentiation using 5×10^6^ hASCs mL^−1^ embedded in collagen bioinks. Both cell constructs were initially cultured in growth media for 7 days and then in differentiation media for up to 3 weeks to assess their potential in promoting muscle tissue regeneration. Conventional bioprinted constructs (control) were fabricated by uniformly mixing hASCs with type I collagen (3 wt.%) and rhodamine (0.2 mm), followed by direct extrusion onto a printing stage maintained at 37 °C to induce thermal‐driven fibrillation, a widely applied process in recent studies.^[^ ^33^ ^]^ The printing parameters were set as follows: pneumatic pressure of 20 kPa, nozzle translation speed of 2 mm s^−1^, barrel temperature of 4 °C, and nozzle diameter of 260 µm.

**Figure 5:**
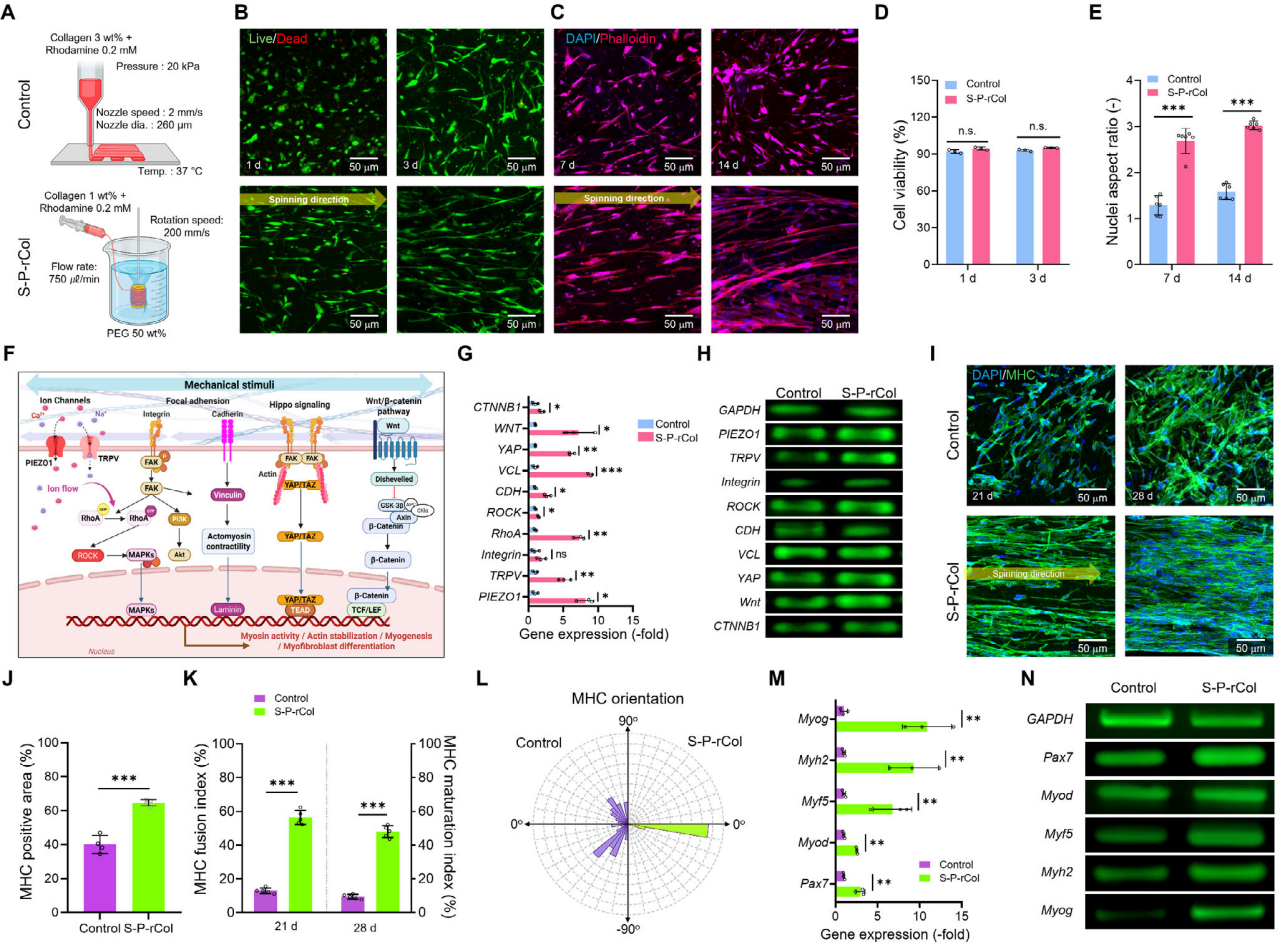
In vitro cellular activities of human adipose‐derived stem cells (hASCs). A) Schematic of the fabrication process for control and stretched collagen crosslinked with rhodamine and polyethylene glycol solution (S‐P‐rCol) bioconstructs. B) Live/dead staining of hASCs on days 1 and 3, and C) DAPI/phalloidin staining on days 7 and 14. D) Quantification of cell viability (*n* = 3) and E) nuclear aspect ratio (*n* = 6). F) Diagram of mechanotransduction pathways involved in muscle differentiation. G) Relative expression levels of mechanotransduction‐related genes (*n* = 3) and H) corresponding agarose gel electrophoresis of PCR products. I) MHC immunofluorescence staining of hASCs on days 21 and 28. Quantification of (J) MHC‐positive area (*n* = 4), K) fusion index (myotubes with ≥2 nuclei), maturation index (myotubes with ≥5 nuclei) (*n* = 5), and L) myotube alignment at day 28. M) Relative expression of myogenesis‐related genes (*n* = 3) and N) corresponding gel electrophoresis results. Data presented as mean ± standard deviation (SD). Statistical significance was determined using Student's t‐test to compare two groups (n.s. = statistical nonsignificance, ^*^ *p*<0.05, ^**^ *p*<0.01, ^***^ *p*<0.001).

In contrast, wet‐spun P‐rCol cell‐laden filaments (S‐P‐rCol) were fabricated under pre‐set conditions (**Figure** **5**A). This approach produced mechanically robust, anisotropic filaments by exposing cells to directional shear stress during extrusion, thereby promoting cytoskeletal reorganization and activating mechanotransduction pathways to facilitate aligned cellular orientation.

To assess cellular viability and alignment, hASCs cultured in both control and S‐P‐rCol bioconstructs were stained using live/dead assays on days 1 and 3, and DAPI/phalloidin on days 7 and 14 (Figure 5B,C). Cell viability measurements indicated that both methods did not compromise initial cell viability (Figure 5D). Notably, the S‐P‐rCol filamentous architecture provided geometric confinement and topographical cues that likely promoted nuclear polarization. The elongated nuclei aligned with the actin filaments reflect cytoskeletal tension, indicating effective biophysical signal transmission through the filament structure. This observation was further supported by orientation factor analysis, which revealed high fiber anisotropy and nuclear elongation along the filament axis in S‐P‐rCol cell filaments (Figure 5E; Figure S19, Supporting Information).

From a mechanobiological perspective, the enhanced alignment and differentiation observed in the S‐P‐rCol structure can be attributed to the application of elongational stress during filament formation, which activates mechanotransduction pathways, such as *YAP*/*TAZ* and *RhoA*/*ROCK*, thereby inducing stem cell fate to myogenesis (Figure 5F).^[^ ^34^, ^35^ ^]^ In addition, the filamentous microenvironment mimics the native ECM architecture of skeletal muscles, which supports the linear organization of myotubes and facilitates force transmission during contraction. To further investigate this effect, the expression levels of mechanotransduction‐related genes (*YAP*, *TAZ*, *RhoA*, *ROCK*, *PIEZO1*, and *TRPV*, etc.) were assessed after three days of culture. RT‐PCR analysis and agarose gel electrophoresis (Figure 5G,H) revealed significantly upregulated gene expression in the S‐P‐rCol group, suggesting enhanced mechanoactivation.

This hypothesis was validated using DAPI/myosin heavy chain (MHC) immunofluorescence staining (Figure 5I). Quantitative analysis showed increased MHC expression in hASCs encapsulated in S‐P‐rCol constructs (Figure 5J). Furthermore, both the MHC fusion index (percentage of myotubes containing ≥2 nuclei) and MHC maturation index (≥ 5 nuclei) were high in the S‐P‐rCol group (Figure 5K), indicating accelerated myogenic differentiation. Furthermore, MHC‐stained images after 21 and 28 days of culture showed elongated, parallel‐aligned actin filaments and extensive MHC expression in the S‐P‐rCol group, whereas the control group showed disorganized cytoskeletal structures and limited MHC expression (Figure 5L).

In addition, the gene expression levels of myogenic markers (*Pax7*, *Myod*, *Myf5*, *Myh2*, and *Myog*) were assessed, and the results demonstrated a significantly elevated expression of the S‐P‐rCol constructs relative to that in the controls (Figure 5M,N).

Generally, rhodamine has been reported to exhibit cytotoxicity at high concentrations, raising concerns about its suitability for biomedical applications.^[^ ^31^, ^36^ ^]^ As such, we optimized the rhodamine concentration to achieve maximal improvement in mechanical properties while minimizing effects on cell viability. In the future, studies should explore alternative bioactive molecules capable of promoting amide bond formation between collagen molecules while maintaining low cytotoxicity and providing functionalities. Promising candidates include naturally derived compounds such as ascorbic acid, heparin, or RGD peptides, which can function under bioorthogonal conditions. These alternatives may not only enhance the long‐term biocompatibility of the constructs but also provide opportunities for further functionalization, such as the incorporation of cell‐instructive signaling.

### 2.6 In Vivo Works

**Figure 6:**
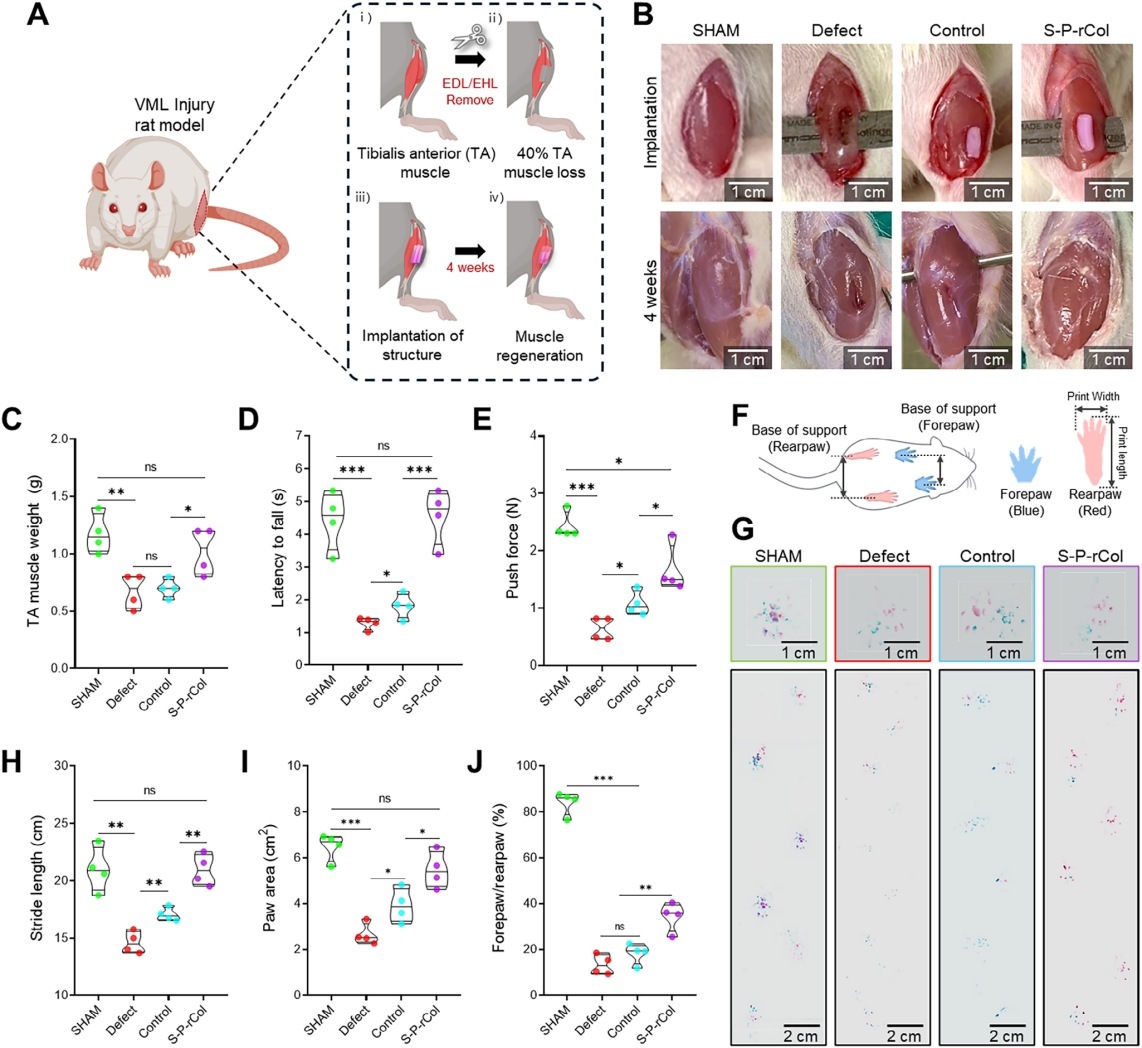
Rat volumetric muscle loss (VML) regenerative efficacy of stretched collagen crosslinked with rhodamine and polyethylene glycol solution (S‐P‐rCol) bioconstructs. A) Schematic illustration of the VML defect induction and bioconstruct implantation procedure. B) Gross images of the defect site immediately after implantation and four weeks post‐implantation. Quantitative assessment of functional recovery at four weeks: C) harvested tibialis anterior (TA) muscle weight, D) latency‐to‐fall, and E) maximum push force. F) Schematic representation and G) optical images of rat footprints used to assess gait patterns. Quantification of gait parameters: H) stride length, I) paw area, and J) forepaw‐to‐rear paw ratio. All data presented as violin plots with individual data points (*n* = 4). Statistical significance was determined using one‐way analysis of variance (ANOVA), followed by Tukey's post‐hoc test (n.s. = statistical nonsignificance, ^*^ *p*<0.05, ^**^ *p*<0.01, ^***^ *p*<0.001).

To further verify the in vitro results, 2×4×1 mm^3^ bioconstructs were implanted directly into VML defects in Sprague Dawley rats. Approximately 40% tibialis anterior (TA) muscles were removed, and the bioconstructs (control and S‐P‐rCol) were placed at the defect site for implantation (**Figure** **6**A,B). Age‐matched (SHAM) and non‐treated (defective) rats were selected as positive and negative comparative groups, respectively, to evaluate the regenerative efficacy of these constructs.

The measured weights of the TA muscle four weeks post‐treatment were 1.2, 0.8, 0.83, and 0.98 g for the SHAM, defect, control construct, and S‐P‐rCol groups, respectively (Figure 6C). These results indicated a notable increase in muscle mass following S‐P‐rCol implantation in the VML model. Figure 6D,E show the evaluation of TA muscle function through latency‐to‐fall, maximum push force, and footprint analyses conducted on live rats four weeks post‐implantation. Both the latency‐to‐fall and push force significantly reduced in all groups one week after the VML injury. However, S‐P‐rCol‐treated rats demonstrated significant functional recovery by week 4, underscoring the therapeutic potential of the bioengineered construct in promoting muscle regeneration. To assess the gait patterns, the forepaws and hind paws were stained with blue and red ink, respectively (Figure 6F), and the animals were allowed to walk across a paper‐covered runway. The resulting footprints (Figure 6G) were quantitatively analyzed for stride length, paw area, and forepaw‐to‐hind paw coverage ratio (Figure 6H–J). Rats with untreated VML defects exhibited significantly reduced stride length and paw area, indicative of an abnormal gait. In contrast, S‐P‐rCol‐treated rats displayed stride lengths and paw areas comparable to those of the SHAM group rats, suggesting restored walking function. Although the forepaw‐to‐hind paw coverage ratio in the S‐P‐rCol group was higher than that in the defect and control groups, it remained lower than that in the SHAM group, indicating partial recovery of coordinated limb movement.

**Figure 7:**
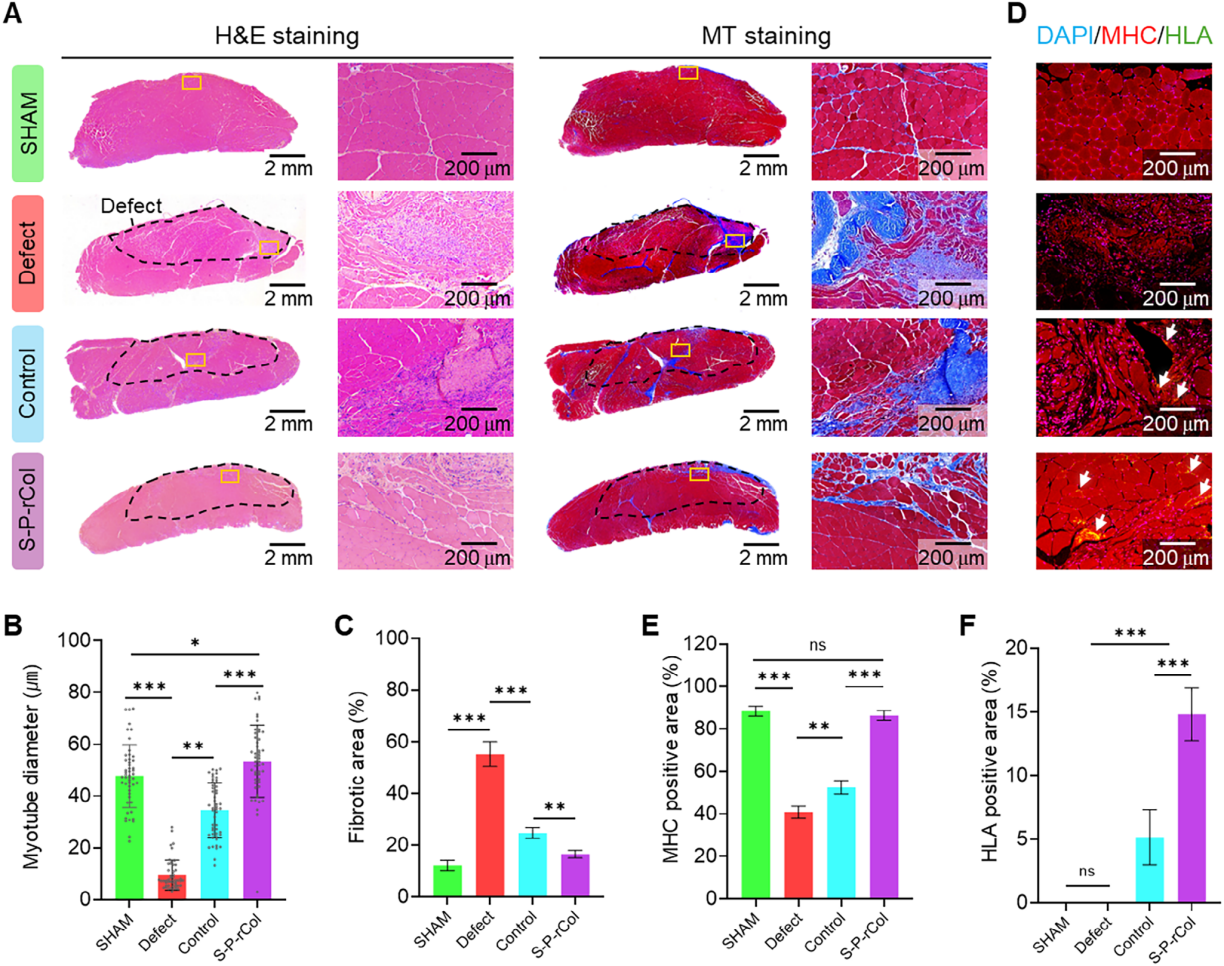
Histological and immunochemical staining evaluation of muscle regeneration. A) H&E and MT histological staining of the implantation site and measured B) myotube diameter (*n* = 50) and C) fibrotic tissue area (*n* = 3). D) Immunochemical staining of DAPI/MHC/HLA of the harvested TA muscle and quantification of E) MHC positive area (*n* = 9) and F) HLA positive area (white arrow indicates HLA expression, *n* = 3). Data presented as mean ± standard deviation (SD). Statistical significance was determined using one‐way analysis of variance (ANOVA), followed by Tukey's post‐hoc test (n.s. = statistical nonsignificance, ^*^ *p*<0.05, ^**^ *p*<0.01, ^***^ *p*<0.001).

Four weeks after implantation, TA muscles were harvested and subjected to histological analyses using Hematoxylin and Eosin (H&E) and Masson's trichrome (MT) staining (**Figure** **7**A). Key parameters, including myofiber diameter and fibrotic tissue extent, were quantitatively analyzed. Compared with the defect group animals, animals treated with bioconstructs exhibited significantly larger muscle fiber diameters, indicating enhanced muscle regeneration. Among all treatment groups, the S‐P‐rCol group demonstrated the largest average fiber diameter, representing a 2‐fold increase relative to the defect group (Figure 7B). In contrast, MT staining revealed extensive fibrotic areas in the defect group, which highlighted impaired regeneration and increased scar tissue formation (Figure 7C). Notably, histological sections from the implanted groups showed reduced fibrotic tissue, suggesting a lower host immune response. These findings are consistent with previous reports demonstrating that hASC‐laden hydrogels can persist and function in rat VML models without strong immune rejection.^[^ ^37^ ^]^ In addition, a study using human muscle progenitor cells and human neural stem cells showed that human cell‐laden constructs could integrate into rodent VML models without eliciting significant immune rejection.^[^ ^38^ ^]^ This is likely attributable to the immunomodulatory properties of hASCs together with the protective microenvironment provided by the hydrogel carrier, which together facilitated host integration and muscle repair.

To characterize the cell types and their origins within the regenerated TA muscle, immunohistochemical staining was conducted using specific markers, including MHC for muscle fibers, human leukocyte antigen (HLA) for human cell identification (Figure 7D). Analyzing the MHC‐positive regions (Figure 7E) demonstrated that the S‐P‐rCol group exhibited markedly higher MHC expression than the defect and control groups, with levels approaching those observed in the SHAM group, indicating enhanced muscle tissue regeneration. As anticipated, the human‐specific markers HLA were not detected in the SHAM and defect groups but expressed in the implant‐treated groups (control and S‐P‐rCol), confirming the presence and survival of implanted hASCs within the host muscle (Figure 7F). Notably, a higher density of HLA‐positive cells was found in the S‐P‐rCol group, suggesting that mechanical stimulation contributed to improved hASC integration into the regenerating muscle environment.

## 3 Conclusion

In this study, we introduce a cytocompatible crosslinking strategy to fabricate structurally reinforced collagen filaments, where the rhodamine‐assisted collagen constructs are referred to as bioorthogonal, achieved through the synergistic combination of rhodamine‐mediated amide bond formation and PEG‐induced dehydration. This method generated highly aligned and mechanically robust protein‐based scaffolds without using cytotoxic catalysts or high protein concentrations. By integrating the crosslinking approach with a wet‐spinning process, we formed uniaxially aligned filaments that can support cell encapsulation and promote mechanosensitive cellular behaviors, such as cytoskeletal organization and myogenic differentiation. In particular, hASC‐laden collagen constructs demonstrated significant regenerative efficacy in a murine VML model, underscoring their potential for clinical translation in muscle tissue engineering. Furthermore, the adaptability of the platform to various protein‐based hydrogels, including gelatin, dECM, and GelMA, highlights its versatility for broader applications in the regeneration of structurally anisotropic tissues. Collectively, our results report the establishment of a scalable and tunable framework for developing mechanically resilient and biologically active scaffolds that can advance the frontiers of regenerative medicine.

## 4 Experimental Section

### Materials

PEG with 6000 g mol^−1^ molecular weight (M_w_) (PEG‐6000), Rhodamine B (83689), N‐Hydroxysuccinimide (NHS; 130672) were purchased from Sigma–Aldrich Inc (St. Louis, MO, USA), while the 1‐Ethyl‐3‐(3‐dimethylaminopropyl)carbodiimide (EDC; D4029) was purchased from Tokyo Chemical Industry (Tokyo, Japan). Additionally, porcine‐derived type I collagen solution was obtained from MSBio (Seungnam, South Korea).

### Bioink Preparation

Type I collagen (10 wt.%) was first dissolved in a 0.1 m acetic acid solution (pH 4.0) and then neutralized by thoroughly mixing with an equal volume of 10× Dulbecco's modified Eagle's medium (DMEM) (Gibco, USA), yielding a 5 wt.% neutralized collagen solution. This solution was further diluted using phosphate‐buffered saline (PBS; final concentration: 1 wt.%). The final bioink was created by incorporating 5×10^6^ cells mL^−1^ murine skeletal muscle cells (C2C12; CRL‐1772, ATCC, Manassas, VA, USA) or hASCs (PT‐5006, Lonza, Basel, Switzerland) into the prepared collagen solution.

For conventional bioprinting applications, a separate collagen solution was prepared by dissolving 10 wt.% type I collagen in 0.1 m acetic acid, followed by neutralization through thorough mixing with an equivalent volume of 10× DMEM, resulting in a 3 wt.% collagen solution. This neutralized collagen solution was loaded with 5×10^6^ hASCs mL^−1^ to form a bioink used for conventional bioprinting experiments.

### Chemical Crosslinking and Fibrillogenesis of Collagen

To evaluate collagen fibrillogenesis under various treatment conditions, 1 wt.% collagen solutions were dispensed into 10×5×2 mm^3^ molds and subsequently treated with 200 mm EDC/NHS solution, DMEM, or 50 wt.% PEG‐6000 solution at 37 °C. After incubation under each condition, the collagen structures were thoroughly rinsed with triple‐distilled water to remove any residual reagents. The same procedure was applied to samples containing rhodamine.

### Collector Design and Fabrication

The spinning collectors used in this study were digitally designed using CAD‐based modeling software (Shapr3D, Shapr3D.Zrt, Hungary) and subsequently exported as standard tessellation language (STL) files. These files were further processed and optimized using slicing software (Official Cubicreator 4 ver. 4.2.4; Cubicon Inc., Seoul, Korea) to prepare them for printing. The finalized collector designs were fabricated by employing a fused filament fabrication (FFF)‐based 3D printing system (Cubicon Single Plus, Cubicon Inc.) using a 1.75 mm‐diameter and 100 µm‐thick PLA filament (PLA‐i21, Cubicon Inc.).

### Wet‐Spinning System for Bioconstruct Fabrication

S‐P‐rCol cell filaments were fabricated using a wet‐spinning system optimized for collagen bioinks. A collagen solution (1 wt.%) containing 5×10^6^ cells mL^−1^ of either hASCs or C2C12 cells was extruded using a syringe pump (Fusion 100; Chemyx, Stafford, TX, USA) at a constant flow rate of 750 µL min^−1^ for 30 s. The extruded collagen solution was directed into a PEG‐6000 coagulation bath maintained at 36 °C, where osmotic dehydration and molecular crowding were induced, leading to rapid fibrillation and solidification of collagen. The resulting hydrogel filaments were continuously collected onto a rotating collector connected to an agitator (HS‐30D; DAIHAN Scientific, South Korea) operating at 200 mm s^−1^. During this process, additional mechanical stretching was applied due to hydrodynamic resistance in the bath, which further enhanced fibril alignment and tensile properties. The collected filaments were highly aligned and accumulated on the collector to form sheet‐like bioconstructs.

As a control, conventional collagen constructs were fabricated via extrusion‐based bioprinting. Specifically, a 3 wt.% collagen bioink containing 5×10^6^ cells mL^−1^ hASCs was dispensed using a pneumatic‐driven 3D bioprinter (DTR3‐2210T‐SG; DASA Robot, South Korea) equipped with a precision dispensing system (AD‐3000C; Ugin‐tech, South Korea). The optimized printing parameters were as follows: dispensing pressure 20 kPa, nozzle diameter 260 µm, printhead speed 2 mm s^−1^, barrel temperature 4 °C, and printing platform temperature 37 °C.

### Mechanical Testing

The mechanical properties of fabricated collagen structures were assessed by tensile testing using a universal testing machine (UTM; Top‐tech, South Korea). Collagen samples (5×10×1.5 mm^3^) were subjected to uniaxial tension at 0.5 mm s^−1^ constant crosshead speed. The tensile modulus was determined from the initial linear region of the stress–strain curves.

### Microscopy

The structural morphology of collagen constructs was examined using optical microscopy (BX FM‐32; Olympus, Japan) coupled with a digital imaging system, as well as SEM (SNE‐3000 M, SEC Inc., South Korea). The acquired images were further processed using ImageJ software for precise dimensional measurements of collagen fibers and to generate corresponding 3D surface maps, providing comprehensive insights into the topographical features of the fabricated collagen structures. For fiber diameter analysis, at least *n* = 25 fibers were randomly selected from three independent constructs per condition, and diameters were quantified using calibrated ImageJ. The diameter distribution was plotted as a violin plot, and the average fiber diameter ± standard deviation (SD) was reported.

### FTIR and Raman Spectroscopy

To assess the degree of collagen fibrillation, FTIR spectroscopy was performed using an FTIR spectrometer (IRTracer‐100, Shimadzu, Kyoto, Japan) over 800–4000 cm^−1^ at 8 cm^−1^ resolution, with each measurement averaged over 30 scans.

Raman spectroscopy was conducted using a Renishaw inVia Raman microscope system (inVia Reflex, Renishaw, Wotton‐under‐Edge, UK) equipped with a 785 nm laser source and ×20 objective lens (Leica DM2700 M, Germany). The SERS spectra were collected at 1.01 mW laser power with 1 s exposure time. For each sample, five accumulations were used for the spectral optimization. Raman spectra were acquired within 800–1400 cm^−1^. All datasets were pre‐processed by removing cosmic ray artifacts and applying polynomial baseline correction using WiRE 5.1 software (Renishaw).

### Ninhydrin Test

A modified ninhydrin‐based colorimetric assay was used to quantify free amine groups. The hydrogels were solubilized in phosphate‐buffered saline (PBS) to a concentration of 10 mg mL^−1^. A freshly prepared 12 mm ninhydrin solution in ethanol was added to each sample at a 1:8 (v/v) ninhydrin: collagen solution. The mixture was incubated at 70 °C for 30 min to enable the reaction between ninhydrin and primary amines. After cooling to room temperature, 100 µL reaction mixture was transferred into a 96‐well plate and the absorbance was recorded at 570 nm using a microplate reader (EL800; BioTek, Vermont, USA). All samples were analyzed in triplicate, and the values were normalized to those of untreated collagen controls.

### Circular Dichroism (CD) Spectroscopy

Circular dichroism spectroscopy (J‐815, JASCO, Japan) was used for analysis of the secondary structure of collagen, particularly focusing on the presence and preservation of the triple‐helical conformation. Collagen, P‐Col, and P‐rCol samples were prepared at a final concentration of 0.2 mg mL^−1^. A 350 µL sample was loaded into a quartz cuvette (1 mm path length; 110‐QS, Hellma, USA), and far‐UV spectra were recorded in the wavelength range of 200–250 nm. All ellipticity measurements were corrected using the buffer spectrum and converted to mean residue ellipticity (MRE) for quantitative comparison.

### In Vitro Cell Culture

Fabricated collagen constructs encapsulating C2C12 myoblasts were cultured in growth medium composed of high‐glucose DMEM (LM 001‐05, Welgene, Gyeongsan‐si, South Korea) supplemented with 10% fetal bovine serum (FBS; Gemini Bio‐Products, USA) and 1% penicillin/streptomycin (P/S; Antimycotic, Thermo Fisher Scientific, USA). To induce the myogenic differentiation of C2C12 cells, differentiation medium containing high‐glucose DMEM supplemented with 2% horse serum (H1270; Sigma–Aldrich, St. Louis, USA) and 1% penicillin was utilized. The bioconstructs containing human adipose‐derived stem cells (hASCs) were initially cultured using low‐glucose DMEM (LM 001‐06; Welgene) supplemented with 10% FBS and 1% penicillin. To promote myogenic differentiation of hASCs, the constructs were subsequently cultured in myogenic induction medium composed of low‐glucose DMEM, 10% FBS, 5% horse serum, 0.1 µm dexamethasone (D4902; Sigma–Aldrich), 50 µm hydrocortisone (H0888; Sigma–Aldrich), and 1% penicillin. Cell‐laden collagen constructs were incubated at 37 °C in a humidified atmosphere containing 5% CO_2_, and the culture medium was replenished every two days throughout the experimental period.

### Cell Viability and Morphological Analyses

Cell viability within the fabricated collagen structures was assessed using live/dead staining. Briefly, the samples were incubated in a staining solution containing 0.15 mm calcein AM and 2 mm ethidium homodimer‐1 (Thermo Fisher Scientific) for 1 h at 37 °C. After staining, confocal microscopy (Carl Zeiss) was used to visualize and quantify live (green) and dead (red) cells. Cell viability was quantitatively analyzed using the ImageJ software (National Institutes of Health, Bethesda, MD, USA).

To investigate cell morphology and cytoskeletal organization, the cultured constructs were fixed with 3.7% formaldehyde (Sigma–Aldrich, 252549) for 1 h, followed by permeabilization with 0.1% Triton X‐100 (Sigma–Aldrich, T8787) for 20 min. The samples were subsequently stained with Alexa Fluor 594‐conjugated phalloidin (1:100 dilution in PBS; Invitrogen, USA) for F‐actin labeling and DAPI (1:100 dilution in PBS; Invitrogen, USA) to visualize nuclei and incubated at room temperature for 90 min. The stained cellular structures were imaged using confocal microscopy (Carl Zeiss), and images were quantitatively analyzed using ImageJ to calculate the orientation factor [= (90°−FWHM)/90°, where FWHM represents the full width at half maximum] and nuclear aspect ratio.

### Immunofluorescence Staining

To evaluate myogenic differentiation within the filament constructs, the samples were first washed three times with PBS, fixed overnight at 4 °C in PBS containing 3.7% formaldehyde, blocked using 2% bovine serum albumin (BSA; Sigma–Aldrich) for 2 h at 37 °C, and permeabilized using PBS containing 2% Triton X‐100 for 2 h at 37 °C. After thorough rinsing with PBS (twice after each incubation step), the samples were incubated overnight at 4 °C with primary antibodies against MHC (MF20; 5 mg mL^−1^; Developmental Studies Hybridoma Bank, Iowa City, IA, USA). The collagen filament‐embedded cells were subsequently incubated with anti‐mouse Alexa Fluor 488‐conjugated secondary antibodies (Invitrogen) diluted 1:50 in PBS for 4 h. Finally, the cell nuclei were counterstained with 5 mm DAPI. The stained filaments were fluorescent imaged using a confocal laser scanning microscope (Carl Zeiss); MHC orientation, fusion index, and maturation index were quantitatively analyzed using ImageJ software.

### Real‐Time Polymerase Chain Reaction

Gene expression levels associated with myogenic differentiation (*Pax7, Myod, Myf5, Myh2*, and *Myog*) and mechanotransduction pathways (*PIEZO1, TRPV, Integrin, CDH, VCL, Wnt, CTNNB1, YAP, TAZ, RhoA, FAK*, and *ROCK*) were determined using quantitative real‐time PCR (qRT‐PCR) and normalized to endogenous housekeeping gene glyceraldehyde‐3‐phosphate dehydrogenase (*GAPDH*) expression. Briefly, total RNA was extracted from cultured samples using TRI reagent (Sigma‐Aldrich), followed by the spectrophotometric evaluation of RNA quality and quantification (FLX800T, BioTek, Winooski, VT, USA). Subsequently, the purified RNA samples were subjected to DNase treatment (RNase‐free), and complementary DNA (cDNA) was synthesized using a reverse transcription kit according to the provided guidelines. qRT‐PCR was conducted using Thunderbird SYBR qPCR Master Mix (Toyobo, Osaka, Japan), and the amplified products were monitored using a real‐time PCR device (StepOnePlus; Applied Biosystems, Foster City, CA, USA), strictly adhering to the manufacturer's recommended protocols. The primer sequences used for gene amplification are listed in Table S2 (Supporting Information).

### Agarose Gel Electrophoresis

The PCR products obtained after 30 cycles were used for DNA electrophoresis and separated on a 1.2% agarose gel. The gel was prepared by dissolving agarose powder in 1× TAE buffer and staining with Loading STAR (Dyne Bio, South Korea). After gelation at room temperature for 30 min, 4 µL of PCR product was mixed with 1 µL of loading buffer and loaded into the wells alongside a DNA ladder. Electrophoresis was conducted at an appropriate voltage until the dye front migrated approximately two‐thirds of the gel length. DNA bands were visualized using a UV transilluminator. To improve the clarity of the bands, ImageJ software was used to adjust the captured images as described in Table S3 (Supporting Information).

### VML Defect Injury Model

To evaluate the muscle regenerative potential of the fabricated constructs, a VML model was established in 8–10 week old Sprague Dawley rats (Central Lab Animal Inc., South Korea), following previously reported protocol.^[^ ^39^ ^]^ Briefly, the rats were housed in separate cages in a local animal laboratory under controlled conditions with unlimited access to rodent food and water following standard laboratory guidelines to optimize animal care. All experimental procedures were conducted under a protocol approved by the Institutional Animal Care and Use Committee (IACUC) of Hallym University (Approval No. 2024‐2‐1209‐44).

Under 3% isoflurane anesthesia, a skin incision was made on the lower left hindlimb, and the extensor digitorum longus and extensor hallucis longus muscles were removed to prevent compensatory hypertrophy. Approximately 40% TA muscle was excised and weighed, with the expected TA weight calculated using the formula: y (g) = 0.0017 × body weight (g) – 0.0716.^[^ ^39^ ^]^ Cell‐laden collagen constructs (1×10^7^ cells mL^−1^, 2×4×1.5 mm^3^) were cultured in growth medium for one day prior to implantation and then implanted into the defect site. The wound was closed by suturing the fascia and skin. The animals were divided into SHAM (positive control), untreated defect (negative control), conventional collagen structure (control), and S‐P‐rCol groups (*n* = 3 per group) and analyzed four weeks post‐implantation.

### Muscle Functional Tests

In vivo muscle function was assessed in live rats using the hanging test and push force evaluation. Briefly, the time taken by each rat to fall from the meshed bar (hang test) was recorded five times per session. For the maximum push force measurement, a force‐sensing platform [47200; Ugo Basile, Germonio (VA), Italy] was used to quantify the peak force generated by the hindlimbs. The rats were gently positioned on the platform, and a gradual manual stimulus was applied to encourage them to push against the calibrated force sensor. Each animal was allowed to acclimate to the setup before testing, and the measurements were repeated five times per session, with adequate rest intervals to minimize fatigue. The highest force recorded in each session was considered the maximum push force. For footprint analysis, the forepaws and rear paws were stained with blue and red ink, respectively. The rats were allowed to walk along a white paper‐covered runway (≈8 cm wide and 100 cm long). The resulting footprints were analyzed using the ImageJ software to assess gait symmetry, stride length, and paw placement.

### Histological and Immunofluorescence Staining

For histological analysis, TA muscle samples were harvested from rats for four weeks post‐implantation, fixed in 3.7% formaldehyde at 25 °C for 24 h, embedded in paraffin, and sectioned at 5 µm thickness. The sections were deparaffinized and subjected to H&E and MT staining to evaluate the general tissue morphology and collagen deposition, respectively. Muscle fiber diameters and collagen‐positive areas were quantified using ImageJ software. For immunofluorescence analysis, the deparaffinized sections were incubated with primary antibodies against mouse MHC (1:50 dilution; Santa Cruz Biotechnology, Dallas, TX, USA) and HLA (1:50 dilution; Abcam, Cambridge, UK). After washing with PBS, the sections were incubated with Alexa Fluor 488‐conjugated anti‐rabbit and Alexa Fluor 594‐conjugated anti‐mouse secondary antibodies (1:200 dilution; Abcam) for 30 min at room temperature. The nuclei were counterstained with DAPI. The stained sections were imaged using a confocal microscope, and the percentage of HLA‐positive myofibers was quantified using ImageJ software.

### Statistical Analysis

All numerical results are presented as mean ± standard deviation (SD), with each experiment performed in triplicate or more (*n* ≥ 3). For normally distributed continuous variables with three or more treatment groups, one‐way analysis of variance (ANOVA) was conducted, followed by Tukey's post‐hoc test for pairwise comparisons. Student's *t*‐test was performed to compare two groups. Statistical analyses were performed using Prism for Windows (version 8.0; GraphPad Prism, Boston, MA, USA). All tests were two‐sided, and the levels of significance are indicated as follows: ^*^ *p*<0.05, ^**^ *p*<0.01, and ^***^ *p*<0.001.

## Conflict of Interest

The authors declare no conflict of interest.

## Author Contributions

J.K. and G.K. conceived and designed the study. J.K. and D.Y. performed in vivo experiments and analysis. J.K., H.H., and B.C. performed in vitro characterization as well as a range of cell and physical experiments. J.K. and G.K. analyzed the data. J.K., H.H., and G.K. drafted the manuscript. G.K. supervised the entire project. All authors discussed the results and commented on the manuscript.

## Supporting information



Supporting Information

Supporting Information

Supporting Information

## Data Availability

The data that support the findings of this study are available from the corresponding author upon reasonable request.
