# Supplementary material for: Catalyst‐Free Collagen Filament Crosslinking for Engineering Anisotropic and Mechanically Robust Tissue Scaffolds
Source: Adv Sci (Weinh). 2025 Nov 18;13(9):e14319. doi: 10.1002/advs.202514319 (PMC12903963; doi:10.1002/advs.202514319)
Supplement: Supplementary file 1 — Supporting Information [file ADVS-13-e14319-s002.docx]

**Supporting Information**

**Catalyst-Free Collagen Filament Crosslinking for Engineering Anisotropic and Mechanically Robust Tissue Scaffolds**

JuYeon Kim,^1^ Hanjun Hwangbo,^1^ ByungJoon Choi,^1^ Dogeon Yoon,^2^ and GeunHyung Kim^1,*^

^1^Department of Precision Medicine, Sungkyunkwan University School of Medicine (SKKU-SOM), Suwon 16419, Republic of Korea

^2^Hangang Sacred Heart Hospital, College of Medicine, Hallym University, Seoul, Republic of Korea

**Supplementary Tables**

**Table S1. Summary of Collagen Crosslinking Methods: Advantages, Disadvantages, and Applications**

| **Crosslinking method** | | **Chemical structure** | **Advantage** | **Disadvantage** | **Application** | **Ref.** |
| --- | --- | --- | --- | --- | --- | --- |
| Chemical | Aldehyde | 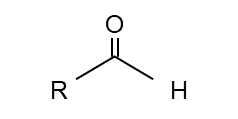 | High mechanical properties and resistance to biodegradation | Signiﬁcant cytotoxicity and biohazard problems | Bone scaffolds/ECM cardiovascular scaffolds | [1-3] |
|  | Isocyanate | 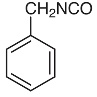 | High mechanical properties and resistance to biodegradation | Cytotoxicity/inﬂammation | Collagen foam/natural polymer synthesis | [4, 5] |
|  | Genipin | 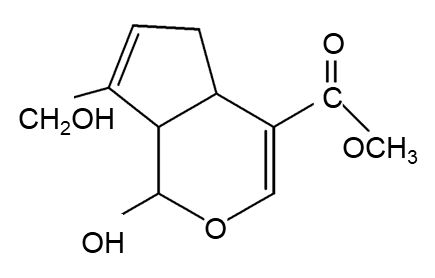 | Biodegradability and low cytotoxicity/inﬂammation | Expensive for mass industrial production | Nanostructured bone scaffolds | [6, 7] |
|  | EDC/NHS | 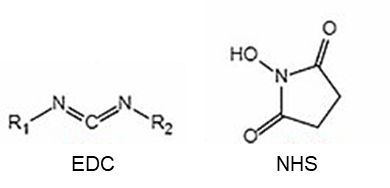 | Water soluble system, low toxicity/inﬂammation | Poor biomechanical properties and more rapid biodegradation proﬁles | Corneal tissue engineering | [8, 9] |
|  | Tanic acid | 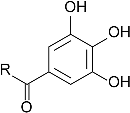 | Nontoxic, potential intrinsic mineralization property | Poor biomechanical properties and more rapid biodegradation proﬁles | Collagen electrospun/collagen sheets | [10, 11] |
| Physical | Plasma treatment | 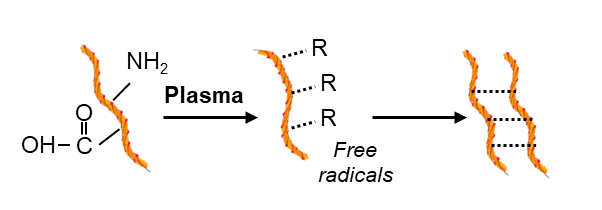 | Simple and safe | Denaturation issues; only acceptable for surface modiﬁcations | Collagen nanoﬁbers | [12, 13] |
|  | UV crosslinking | 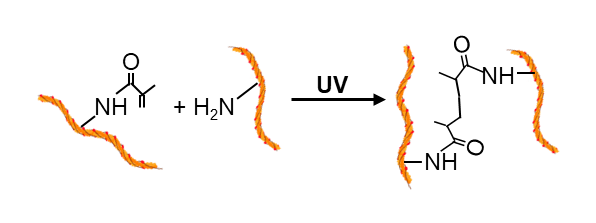 | Nontoxic | Denaturation issues | Collagen ﬁbers/corneal engineering | [14-17] |
|  | Dehydrothermal treatment | 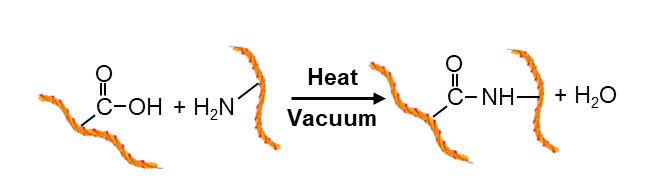 | Simple and safe | Denaturation issues; require further modiﬁcation | Electrospun collagen | [18, 19] |
| Biological | Transglutaminase | 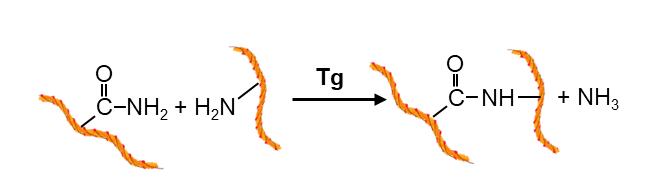 | Nontoxic | Expensive; low stability | Modiﬁed collagen ﬁbers | [20-22] |

**EDC/NHS**, 1-ethyl-3-(3-dimethylaminopropyl) carbodiimide/N-hydroxysuccinimide; **UV**, ultraviolet; **ECM**, extracellular matrix; **Tg**, transglutaminase.

**Table S2. Gene primer sequences used in the qRT-PCR analysis.**

| Gene | Source | Primer sequence | |
| --- | --- | --- | --- |
|  |  | Left (5ʹ – 3ʹ) | Right (5ʹ – 3ʹ) |
| *GAPDH* | *Homo sapiens* | TTTTGCGTCGCCAGCC | TTTTGCGTCGCCAGCC |
| *PIEZO1* | *Homo sapiens* | ACTTTCCCATCAGCACTCGG | AGGGTGTAGAGCAACATGGC |
| *TRPV* | *Homo sapiens* | CTCACCTGAAAGCGGAGGTT | AGAGGCACCATCCTCATCCT |
| *Integrin* | *Homo sapiens* | CGAAAGTGCCATGCGATCTG | GCCATGGGGTCCAAGTACAC |
| *CDH* | *Homo sapiens* | TGGGCCAGGAAATCACATCC | TGCAACGTCGTTACGAGTCA |
| *VCL* | *Homo sapiens* | TGAGCAAGCACAGCGGTGGATT | TCGGTCACACTTGGCGAGAAGA |
| *Wnt* | *Homo sapiens* | CCGCAACTATAAGAGGCGGT | AAGGTTCATGAGGAAGCGCA |
| *CTNNB1* | *Homo sapiens* | GGCTACTCAAGCTGATTTGATGG | GCAGGAATGCCTCCAGACTT |
| *YAP* | *Homo sapiens* | CCCTCGTTTTGCCATGAACC | AATTCAGTCTGCCTGAGGGC |
| *TAZ* | *Homo sapiens* | AGCCCTTTCTAACCTGGCTG | TGACTAATGCTGCTGCTGCT |
| *RhoA* | *Homo sapiens* | gtccacggtctggtcttcag | cagccattgctcaggcaac |
| *FAK* | *Homo sapiens* | GCTCCCTTGCATCTTCCAGT | ATTGCAGCCCTTGTCCGTTA |
| *ROCK* | *Homo sapiens* | TGAAAGCCGCACTGATGGAT | AAGCAGCTCTCCTGGTTGAC |
| *Pax7* | *Homo sapiens* | GAGGACCAAGCTGACAGAGG | GGGTGGAGAGGCTCACATTTT |
| *Myod* | *Homo sapiens* | CGACGGCATGATGGACTACA | CCCTCAAGGTTCAGCTCTGG |
| *Myf5* | *Homo sapiens* | GGGTGAATTTGGGGACGAGT | TGCCATCAGAGCAGTTGGAG |
| *Myh2* | *Homo sapiens* | TCCTGCTTTAAAAAGCTCCAAGAA | ACGCTTGGTGTTCACAGTCT |
| *Myog* | *Homo sapiens* | TCCATCGTGGACAGCATCAC | ACAGGAGACCTTGGTCGGA0054 |
| *GAPDH* | *Mus musculus* | GCCTCCTCCAATTCAACCCT | CTCGTGGTTCACACCCATCA |
| *PIEZO1* | *Mus musculus* | GTCTGTGACAGTCCGTTTGT | CCAGTCAGTGGTCATTCCCC |
| *YAP* | *Mus musculus* | CCTCGTTTTGCCATGAACCA | AACCAACGTAAGAGCAGCGA |
| *TAZ* | *Mus musculus* | TGGGAAGTGGTGGTACAGGA | GTTTGTGAGCAGTGGGCAAG |
| *RhoA* | *Mus musculus* | TTCCCACGTCTACTAGCTTGCAG | CGTTCTTGAGCAATCGTGGC |
| *FAK* | *Mus musculus* | ATTGCAACAGCCAAAGCTGG | CGGACACATGCAGTCTCTGT |
| *ROCK* | *Mus musculus* | TGAAAGCCGCACTGATGGAT | TGCCATCTATTCATTCCAGCCA |
| *MAPK1* | *Mus musculus* | TCAAGCCTTCCAACCTCCTGCT | AGCTCTGTACCAACGTGTGGCT |
| *Pxn* | *Mus musculus* | GTGAGAAGGACTACCACAGCCT | GGACCAAAGAAGGCTCCACACT |

***GAPDH***, glyceraldehyde-3-phosphate dehydrogenase; ***PIEZO1***, piezo-type mechanosensitive ion channel component 1; ***TRPV***, Transient Receptor Potential Vanilloid; ***CDH***, cadherin; ***VCL***, vinculin; ***Wnt***, wingless and Int-1; ***CTNNB1***, β-catenin; ***YAP***, yes-associated protein 1; ***TAZ***, tafazzin; ***RhoA***, ras homolog family member A; ***FAK***, focal adhesion kinase; ***ROCK***, rho-associated coiled-coil containing protein kinase; ***Pax7***, paired box 7; ***Myod***, myogenic differentiation 1; ***Myf5***, myogenic factor 5; ***Myh2***, myosin heavy chain 2; ***Myog***, myogenin; ***MAPK1***, mitogen-activated protein kinase 1; ***Pxn***, paxillin.

**Table S3. Image processing for:**

**Figure 3H**

| Gene | Original data | Image transformation | Treated data |
| --- | --- | --- | --- |
| *GAPDH* | 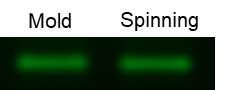 | 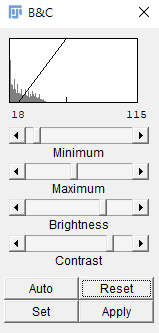  Software: ImageJ  Adjust mode: Brightness/Contrast...  (Scale: 0 ~ 255)  Minimum: 18  Maximum: 115 | 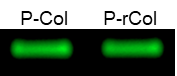 |
| *YAP* | 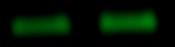 | 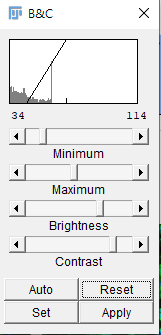  Software: ImageJ  Adjust mode: Brightness/Contrast...  (Scale: 0 ~ 255)  Minimum: 34  Maximum: 114 | 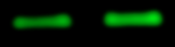 |
| *TAZ* | 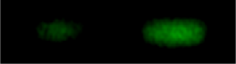 | 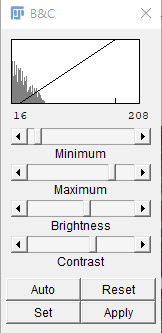  Software: ImageJ  Adjust mode: Brightness/Contrast...  (Scale: 0 ~ 255)  Minimum: 16  Maximum: 208 | 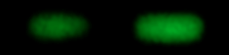 |
| *RhoA* | 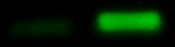 | 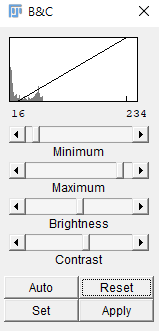  Software: ImageJ  Adjust mode: Brightness/Contrast...  (Scale: 0 ~ 255)  Minimum: 16  Maximum: 234 | 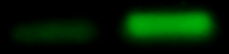 |
| *FAK* | 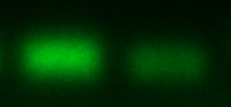 | 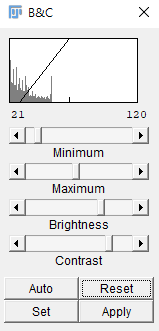  Software: ImageJ  Adjust mode: Brightness/Contrast...  (Scale: 0 ~ 255)  Minimum: 21  Maximum: 120 | 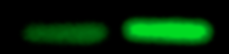 |
| *ROCK* | 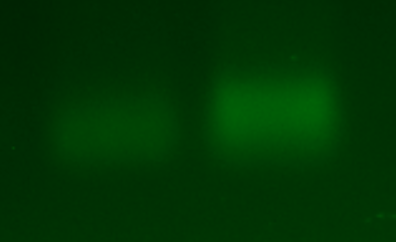 | 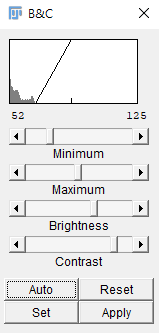  Software: ImageJ  Adjust mode: Brightness/Contrast...  (Scale: 0 ~ 255)  Minimum: 52  Maximum:125 | 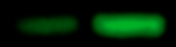 |

**Figure 4D**

| Gene | Original data | Image transformation | Treated data |
| --- | --- | --- | --- |
| *GAPDH* | 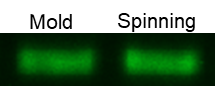 | 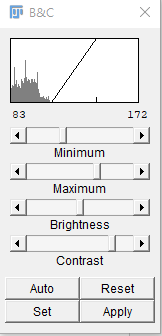  Software: ImageJ  Adjust mode: Brightness/Contrast...  (Scale: 0 ~ 255)  Minimum: 83  Maximum: 172 | 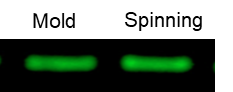 |
| *RhoA* | 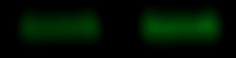 | 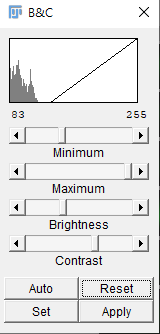  Software: ImageJ  Adjust mode: Brightness/Contrast...  (Scale: 0 ~ 255)  Minimum: 83  Maximum: 255 | 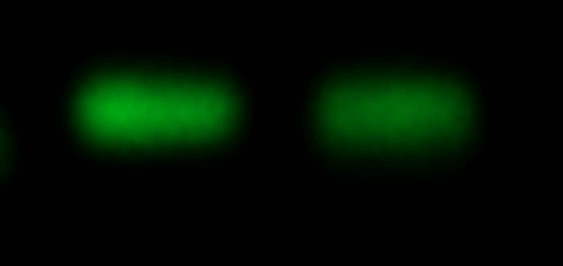 |
| *MAPK1* | 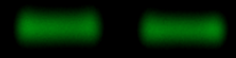 | 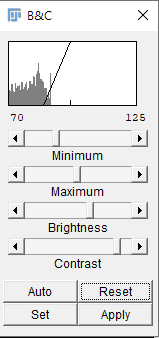  Software: ImageJ  Adjust mode: Brightness/Contrast...  (Scale: 0 ~ 255)  Minimum: 70  Maximum: 125 | 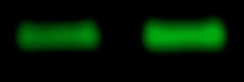 |
| *ROCK* | 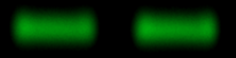 | 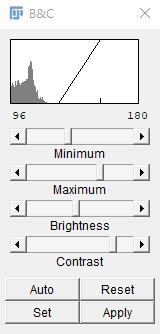  Software: ImageJ  Adjust mode: Brightness/Contrast...  (Scale: 0 ~ 255)  Minimum: 96  Maximum: 180 | 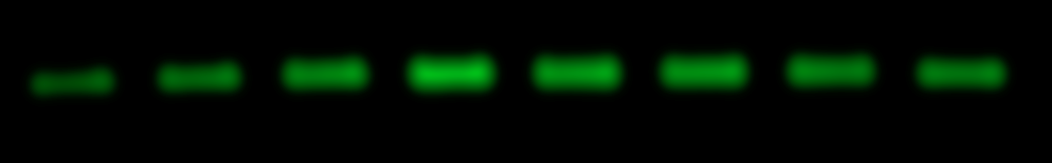 |
| *YAP* | 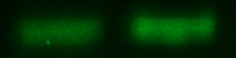 | 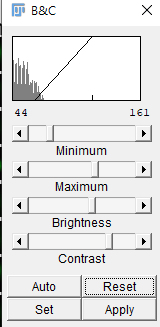  Software: ImageJ  Adjust mode: Brightness/Contrast...  (Scale: 0 ~ 255)  Minimum: 44  Maximum: 161 | 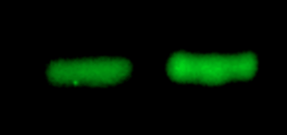 |
| *PIEZO1* | 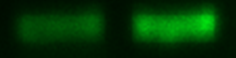 | 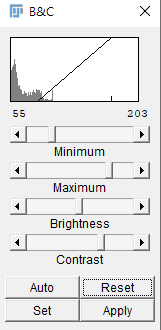  Software: ImageJ  Adjust mode: Brightness/Contrast...  (Scale: 0 ~ 255)  Minimum: 55  Maximum: 203 | 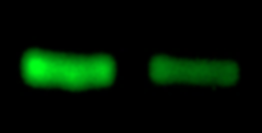 |

**Figure 5H**

| Gene | Original data | Image transformation | Treated data |
| --- | --- | --- | --- |
| *GAPDH* | 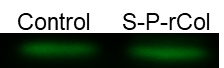 | 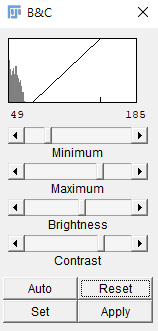  Software: ImageJ  Adjust mode: Brightness/Contrast...  (Scale: 0 ~ 255)  Minimum: 2  Maximum: 130 | 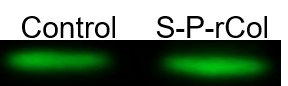 |
| *PIEZO1* | 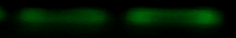 | 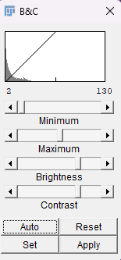  Software: ImageJ  Adjust mode: Brightness/Contrast...  (Scale: 0 ~ 255)  Minimum: 8  Maximum: 141 | 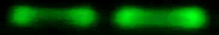 |
| *TRPV* | 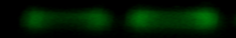 | 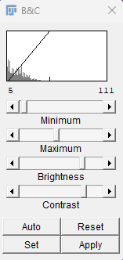  Software: ImageJ  Adjust mode: Brightness/Contrast...  (Scale: 0 ~ 255)  Minimum: 5  Maximum: 111 | 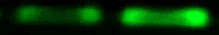 |
| *Integrin* | 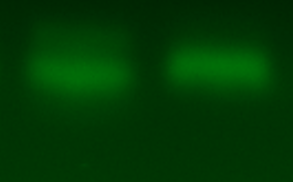 | 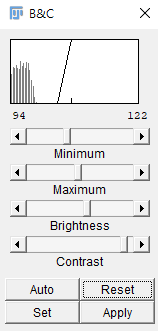  Software: ImageJ  Adjust mode: Brightness/Contrast...  (Scale: 0 ~ 255)  Minimum: 94  Maximum: 122 | 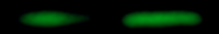 |
| *ROCK* | 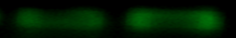 | 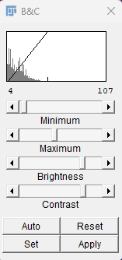  Software: ImageJ  Adjust mode: Brightness/Contrast...  (Scale: 0 ~ 255)  Minimum: 4  Maximum: 107 | 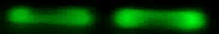 |
| *CDH* | 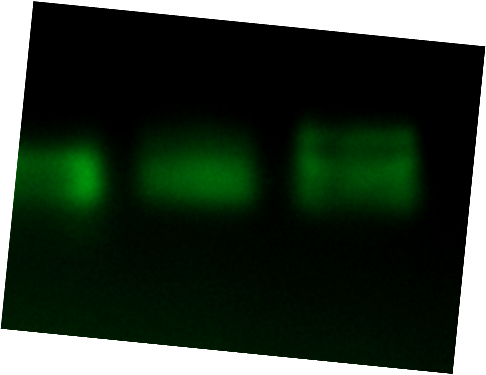 | 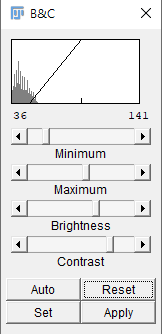  Software: ImageJ  Adjust mode: Brightness/Contrast...  (Scale: 0 ~ 255)  Minimum: 36  Maximum: 141 | 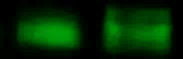 |
| *VCL* | 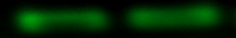 | 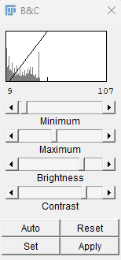  Software: ImageJ  Adjust mode: Brightness/Contrast...  (Scale: 0 ~ 255)  Minimum: 9  Maximum: 107 | 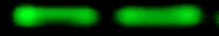 |
| *YAP* | 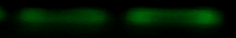 | 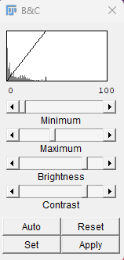  Software: ImageJ  Adjust mode: Brightness/Contrast...  (Scale: 0 ~ 255)  Minimum: 39  Maximum: 105 | 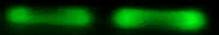 |
| *Wnt* | 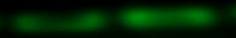 | 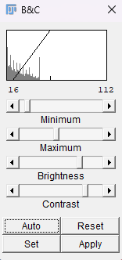  Software: ImageJ  Adjust mode: Brightness/Contrast...  (Scale: 0 ~ 255)  Minimum: 0  Maximum: 100 | 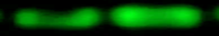 |
| *CTNNB1* | 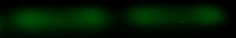 | 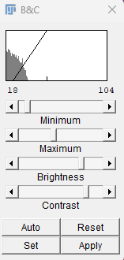  Software: ImageJ  Adjust mode: Brightness/Contrast...  (Scale: 0 ~ 255)  Minimum: 18  Maximum: 104 | 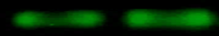 |

**Figure 5N**

| Gene | Original data | Image transformation | Treated data |
| --- | --- | --- | --- |
| *GAPDH* | 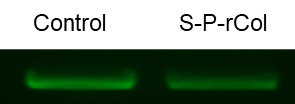 | 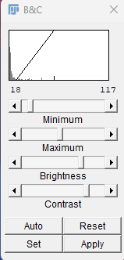  Software: ImageJ  Adjust mode:  Brightness/Contrast...  (Scale: 0 ~ 255)  Minimum: 18  Maximum: 117 | 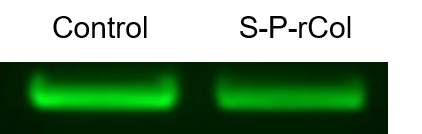 |
| *Pax7* | 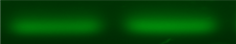 | 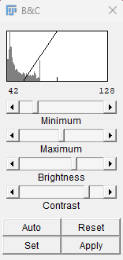  Software: ImageJ  Adjust mode:  Brightness/Contrast...  (Scale: 0 ~ 255)  Minimum: 42  Maximum: 128 | 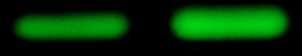 |
| *Myod* | 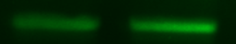 | 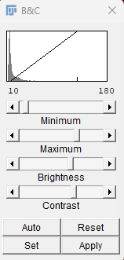  Software: ImageJ  Adjust mode:  Brightness/Contrast...  (Scale: 0 ~ 255)  Minimum: 10  Maximum: 180 | 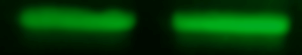 |
| *Myf5* | 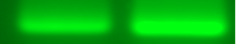 | 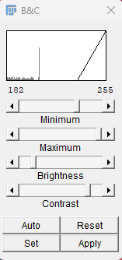  Software: ImageJ  Adjust mode: Brightness/Contrast...  (Scale: 0 ~ 255)  Minimum: 182  Maximum: 255 | 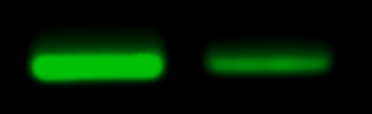 |
| *Myh2* | 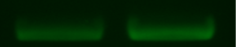 | 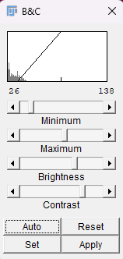  Software: ImageJ  Adjust mode: Brightness/Contrast...  (Scale: 0 ~ 255)  Minimum: 26  Maximum: 138 | 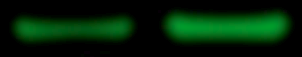 |
| *Myog* | 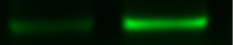 | 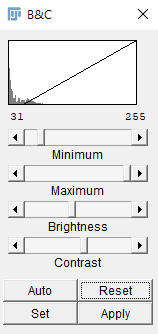  Software: ImageJ  Adjust mode: Brightness/Contrast...  (Scale: 0 ~ 255)  Minimum: 31  Maximum: 255 | 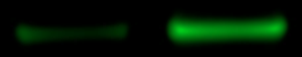 |

**References**

1. Oryan, A.; Kamali, A.; Moshiri, A.; Baharvand, H.; Daemi, H., "Chemical crosslinking of biopolymeric scaffolds: Current knowledge and future directions of crosslinked engineered bone scaffolds," *International journal of biological macromolecules* (2018): *107,* 678-688

2. Ma, B.; Wang, X.; Wu, C.; Chang, J., "Crosslinking strategies for preparation of extracellular matrix-derived cardiovascular scaffolds," *Regenerative biomaterials* (2014): *1,* 1, 81-89

3. Olde Damink, L.; Dijkstra, P.; Van Luyn, M.; Van Wachem, P.; Nieuwenhuis, P.; Feijen, J., "Glutaraldehyde as a crosslinking agent for collagen-based biomaterials," *Journal of materials science: materials in medicine* (1995): *6,* 460-472

4. Gögele, C.; Hahn, J.; Elschner, C.; Breier, A.; Schröpfer, M.; Prade, I.; Meyer, M.; Schulze-Tanzil, G., "Enhanced growth of lapine anterior cruciate ligament-derived fibroblasts on scaffolds embroidered from poly (l-lactide-co-ε-caprolactone) and polylactic acid threads functionalized by fluorination and hexamethylene diisocyanate cross-linked collagen foams," *International Journal of Molecular Sciences* (2020): *21,* 3, 1132

5. Hassan, R. M., "Novel synthesis of natural cation exchange resin by crosslinking the sodium alginate as a natural polymer with 1, 6-hexamethylene diisocyanate in inert solvents: Characteristics and applications," *International Journal of Biological Macromolecules* (2021): *184,* 926-935

6. Montalbano, G.; Borciani, G.; Cerqueni, G.; Licini, C.; Banche-Niclot, F.; Janner, D.; Sola, S.; Fiorilli, S.; Mattioli-Belmonte, M.; Ciapetti, G., "Collagen hybrid formulations for the 3d printing of nanostructured bone scaffolds: An optimized genipin-crosslinking strategy," *Nanomaterials* (2020): *10,* 9, 1681

7. Wang, Z.; Liu, H.; Luo, W.; Cai, T.; Li, Z.; Liu, Y.; Gao, W.; Wan, Q.; Wang, X.; Wang, J., "Regeneration of skeletal system with genipin crosslinked biomaterials," *Journal of Tissue Engineering* (2020): *11,* 2041731420974861

8. Yang, C., "Enhanced physicochemical properties of collagen by using EDC/NHS-crosslinking," *Bulletin of Materials Science* (2012): *35,* 913-918

9. Goodarzi, H.; Jadidi, K.; Pourmotabed, S.; Sharifi, E.; Aghamollaei, H., "Preparation and in vitro characterization of cross-linked collagen–gelatin hydrogel using EDC/NHS for corneal tissue engineering applications," *International journal of biological macromolecules* (2019): *126,* 620-632

10. Cumming, M. H.; Leonard, A. R.; LeCorre-Bordes, D. S.; Hofman, K., "Intra-fibrillar citric acid crosslinking of marine collagen electrospun nanofibres," *International journal of biological macromolecules* (2018): *114,* 874-881

11. Andonegi, M.; de la Caba, K.; Guerrero, P., "Effect of citric acid on collagen sheets processed by compression," *Food Hydrocolloids* (2020): *100,* 105427

12. Prasertsung, I.; Damrongsakkul, S.; Saito, N., "Crosslinking of a gelatin solutions induced by pulsed electrical discharges in solutions," *Plasma Processes and Polymers* (2013): *10,* 9, 792-797

13. Liguori, A.; Bigi, A.; Colombo, V.; Focarete, M. L.; Gherardi, M.; Gualandi, C.; Oleari, M. C.; Panzavolta, S., "Atmospheric pressure non-equilibrium plasma as a green tool to crosslink gelatin nanofibers," *Scientific reports* (2016): *6,* 1, 38542

14. Davidenko, N.; Bax, D. V.; Schuster, C. F.; Farndale, R. W.; Hamaia, S. W.; Best, S. M.; Cameron, R. E., "Optimisation of UV irradiation as a binding site conserving method for crosslinking collagen-based scaffolds," *Journal of Materials Science: Materials in Medicine* (2016): *27,* 1-17

15. Weadock, K. S.; Miller, E. J.; Bellincampi, L. D.; Zawadsky, J. P.; Dunn, M. G., "Physical crosslinking of collagen fibers: comparison of ultraviolet irradiation and dehydrothermal treatment," *Journal of biomedical materials research* (1995): *29,* 11, 1373-1379

16. Bax, D. V.; Davidenko, N.; Hamaia, S. W.; Farndale, R. W.; Best, S. M.; Cameron, R. E., "Impact of UV-and carbodiimide-based crosslinking on the integrin-binding properties of collagen-based materials," *Acta biomaterialia* (2019): *100,* 280-291

17. Nagaraj, S.; Easwaramoorthi, S.; Rao, J. R.; Thanikaivelan, P., "Probing visible light induced photochemical stabilization of collagen in green solvent medium," *International Journal of Biological Macromolecules* (2019): *131,* 779-786

18. Ma, X. H.; Noishiki, Y.; Yamane, Y.; Iwai, Y.; Marato, D.; Matsumoto, A., "Thermal cross-linking for biologically degradable materials: preliminary report," *Asaio Journal* (1996): *42,* 5, M866-870

19. Drexler, J. W.; Powell, H. M., "Dehydrothermal crosslinking of electrospun collagen," *Tissue Engineering Part C: Methods* (2011): *17,* 1, 9-17

20. Cheng, S.; Wang, W.; Li, Y.; Gao, G.; Zhang, K.; Zhou, J.; Wu, Z., "Cross-linking and film-forming properties of transglutaminase-modified collagen fibers tailored by denaturation temperature," *Food chemistry* (2019): *271,* 527-535

21. Liu, Y.; Weng, R.; Wang, W.; Wei, X.; Li, J.; Chen, X.; Liu, Y.; Lu, F.; Li, Y., "Tunable physical and mechanical properties of gelatin hydrogel after transglutaminase crosslinking on two gelatin types," *International journal of biological macromolecules* (2020): *162,* 405-413

22. Olde Damink, L.; Dijkstra, P.; Van Luyn, M.; Van Wachem, P.; Nieuwenhuis, P.; Feijen, J., "Crosslinking of dermal sheep collagen using hexamethylene diisocyanate," *Journal of Materials Science: Materials in Medicine* (1995): *6,* 429-434

**Supplementary Figures**

**
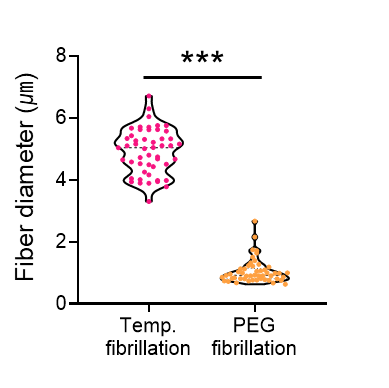
**

**Supplementary Figure S1. Effect of crosslinking method on collagen fiber diameter.** Data presented as violin plots with individual data points (n = 50). Statistical significance was determined using Student’s t-test (***p<0.001).

**
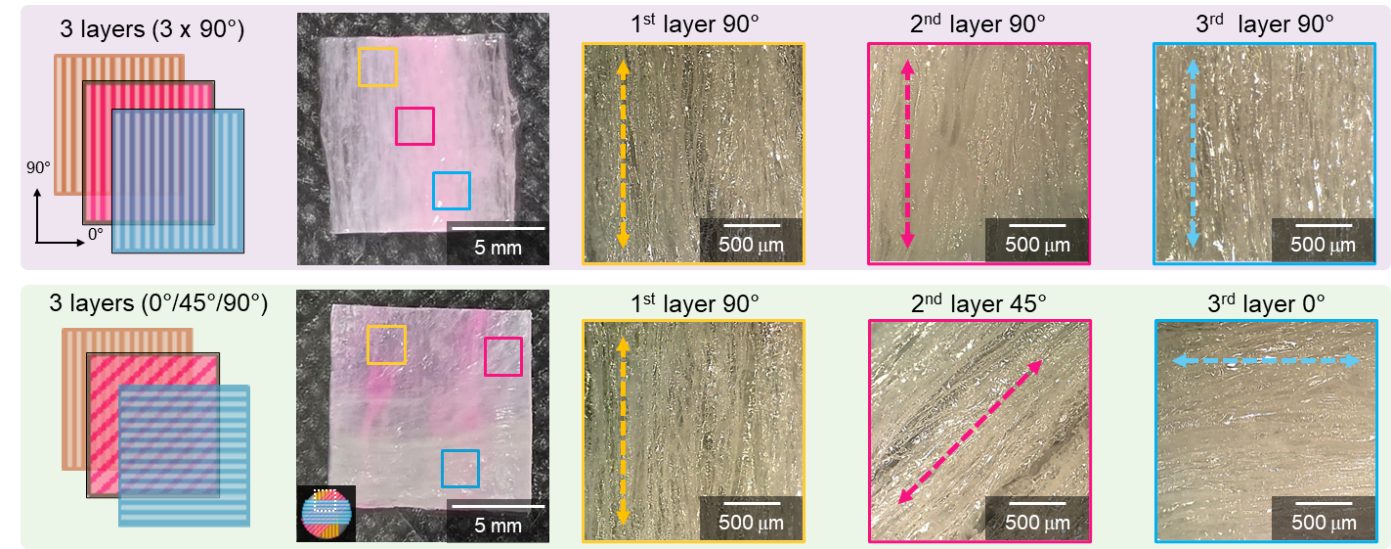
 Supplementary Figure S2. Optical images of multilayered constructs with an anisotropic structure (3 × 90°) and an alternating structure (0°/45°/90°).**

**
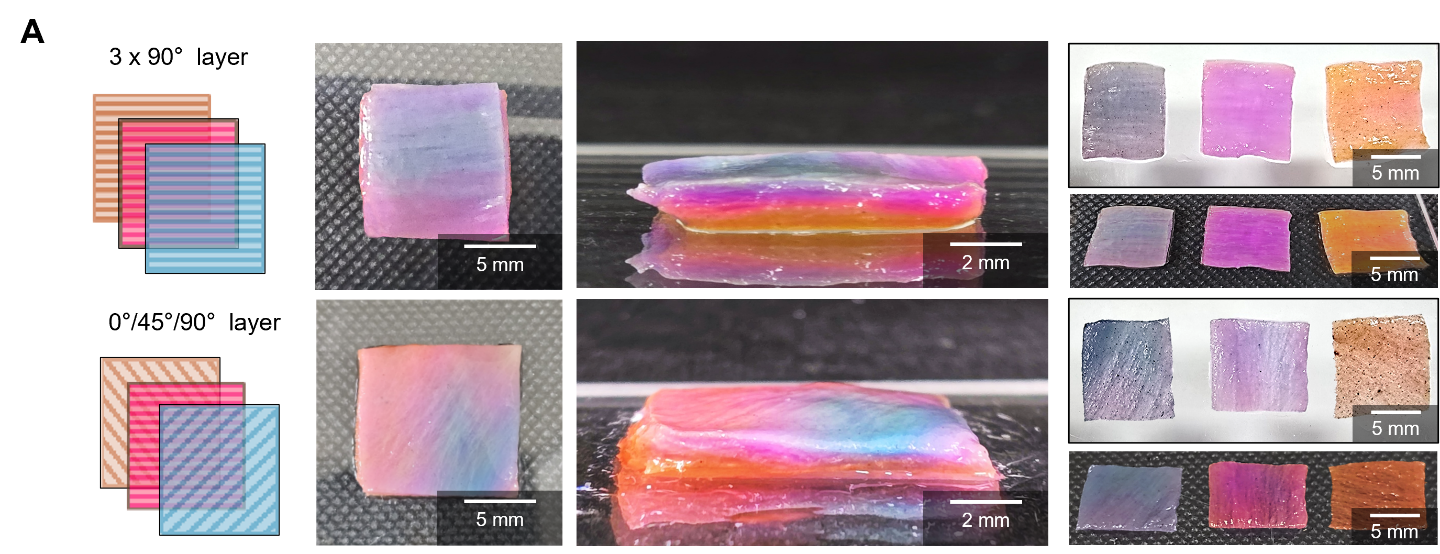
**

**Supplementary Figure S3. Multilayered constructs with each layer containing dyes orange (1^st^ layer), magenta (2^nd^ layer), and violet (3^rd^ layer).**

**
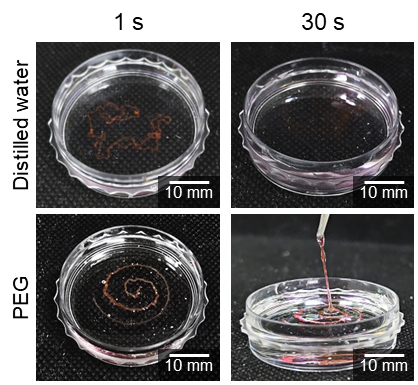
**

**Supplementary Figure S4. Optical images showing the extrusion of rhodamine-incorporated Col hydrogel into distilled water and a PEG bath, demonstrating enhanced structural integrity in PEG.** **
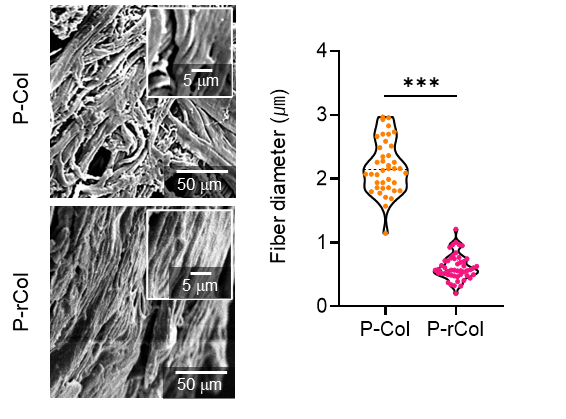
**

**Supplementary Figure S5. Scanning electron microscopy (SEM) images with corresponding measurements of fiber diameter for Col dehydrated with PEG solution (P-Col) and Col crosslinked with rhodamine and PEG solution (P-rCol).** Data presented as violin plots with individual data points (n = 40). Statistical significance was determined using Student’s t-test (***p<0.001).

**
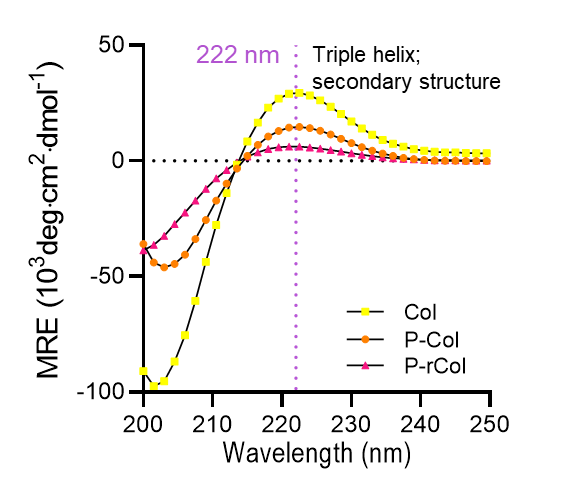
**

**Supplementary Figure S6. Circular dichroism (CD) spectroscopy results of collagen, P-col and P-rCol demonstrating mean residue ellipticity (MRE).**

**
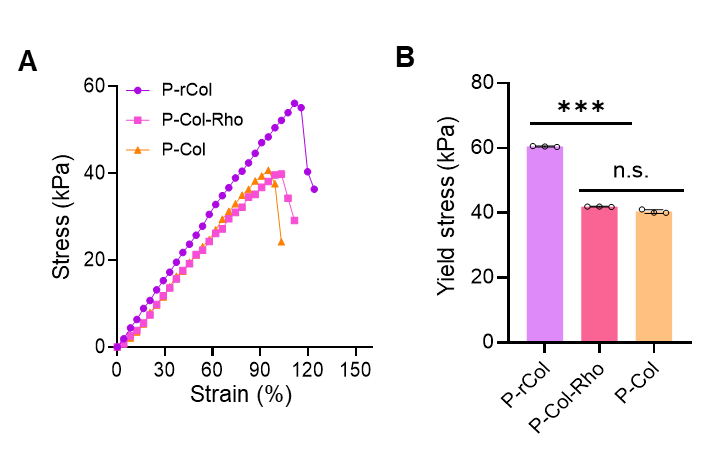
**

**Supplementary Figure S7. Mechanical properties of various collagen-based structures.** (**A**) Stress–strain curves and (**B**) quantified tensile modulus and yield stress for P-Col, P-Col-Rho, and P-rCol structures (n = 3). Data presented as mean ± standard deviation (SD). Statistical significance was determined using one-way ANOVA with Tukey’s post-hoc test (n.s. = statistical nonsignificance, ***p<0.001).

**Supplementary Figure S8. Effects of wet spinning on cellular alignment.** (**A**) Nuclei aspect ratio (n = 26) and (**B**) F-actin orientation distribution of cells cultured in collagen structures fabricated inside a mold and the wet-spinning process. Data presented as mean ± standard deviation (SD). Statistical significance was determined using Student’s t-test (***p<0.001).

**Supplementary Figure S9. Optimization process of the wet-spinning system.** (**A**) Schematics demonstrating parameters of the wet-spinning process. (**B**) Process diagram for rhodamine-assisted wet-spinning at various collagen concentrations and collector rotational speeds.

**Supplementary Figure S10.** Tensile modulus (n = 6). Data presented as mean ± standard deviation (SD). Statistical significance was determined using one-way ANOVA with Tukey’s post-hoc test (***p<0.001).

**Supplementary Figure S11. Live/Dead staining images of cells exposed to rhodamine concentrations (0.1 and 0.8 mM) and crosslinking time (10 and 120 s).**

**Supplementary Figure S12. Effects of wet spinning and rhodamine addition on collagen fiber morphology and mechanical property.** (**A**) Scanning electron microscope (SEM) images and 3D surface topography of stretched collagen crosslinked with polyethylene glycol (PEG) solution (S-P-Col) and stretched collagen crosslinked with rhodamine and PEG solution (S-P-rCol). (**B**) Measured collagen fiber diameters (n = 50). (**C**) Stress–strain curves of various bioconstructs including S-P-rCol, S-P-Col, M-P-rCol (collagen mixed with rhodamine and crosslinked with PEG in a mold) and M-P-Col (collagen crosslinked with PEG in a mold). Data presented as violin plots with individual data points. Statistical significance was determined using Student’s t-test (***p<0.001).

**Supplementary Figure S13. S-P-rCol morphological assessment.** (**A**) Optical images and (**B**) surface SEM image of the fabricated constructs.

**Supplementary Figure S14. Degradation and mechanical strength assessment of S-P-rCol constructs.** (**A**) Degradation profiles of S-P-rCol constructs immersed in PBS and collagenase (10 U/mL). (**B**) Representative stress–strain curves of constructs incubated in collagenase (10 U/mL). (**C**) Quantification of tensile modulus over the degradation period (n = 3). Data presented as mean ± standard deviation (SD). Statistical significance was determined using one-way ANOVA with Tukey’s post-hoc test (***p<0.001).

**Supplementary Figure S15. Assessment of various rhodamine conditions on physical properties of wet-spun structures.** (**A**) Schematic diagram. (**B**) Optical microscopy, SEM images, and 3D surface mapping of samples with different rhodamine concentrations. (**C**) Tensile modulus (n = 3) and (**D**) yield stress as a function of rhodamine concentration (n = 3). (**E**) Dependence of tensile modulus on fiber diameter. Data presented as mean ± standard deviation (SD). Statistical significance was determined using one-way ANOVA with Tukey’s post-hoc test (***p<0.001).

**Supplementary Figure S16. Assessment of various rhodamine conditions on the physical properties of wet-spun structures.** (**A**) Optical microscopy, SEM images, and 3D surface mapping of samples with different rhodamine concentrations. (**B**) Circularity (n = 3) and (**C**) height as a function of rhodamine concentration (n = 3). Data presented as mean ± standard deviation (SD). Statistical significance was determined using one-way ANOVA with Tukey’s post-hoc test (n.s. = statistical nonsignificance, *p<0.05, **p<0.01, ***p<0.001).

**Supplementary Figure S17. Optical images of the hanging weight test according to rhodamine concentration.**

**Supplementary Figure S18. FTIR spectra of collagen constructs with varying rhodamine concentrations.**

**Supplementary Figure S19. F-actin orientation distribution of hASCs cultured in control and S-P-rCol bioconstructs at day 14.**

**Supplementary Movie 1**

PEG/rhodamine-assisted spinning process (Fig. 2A).

**Supplementary Movie 2**

A hanging weight test using a 12g load of collagen constructs containing 0.1 and 1.6 mM of rhodamine (Fig. 4K).
